# Brown Adipose Tissue undergoes pathological perturbations and shapes C2C12 myoblast homeostasis in the SOD1-G93A mouse model of Amyotrophic Lateral Sclerosis

**DOI:** 10.1016/j.heliyon.2025.e41801

**Published:** 2025-01-23

**Authors:** Marco Rosina, Silvia Scaricamazza, Flaminia Riggio, Gianmarco Fenili, Flavia Giannessi, Alessandro Matteocci, Valentina Nesci, Illari Salvatori, Daniela F. Angelini, Katia Aquilano, Valerio Chiurchiù, Daniele Lettieri Barbato, Nicola Biagio Mercuri, Cristiana Valle, Alberto Ferri

**Affiliations:** a Unit of Neurology, Fondazione PTV Policlinico Tor Vergata, Viale Oxford 81, 00133, Rome, Italy; b Laboratory of Neurochemistry, IRCCS Fondazione Santa Lucia, Via del fosso di fiorano 64, 00143, Rome, Italy; c Institute of Translational Pharmacology, National Research Council, Via del fosso del cavaliere 100, 00133, Rome, Italy; d Department of Biology and Biotechnology “Charles Darwin”, University of Roma “La Sapienza”, 00161, Rome, Italy; e Department of Movement, Human and Health Sciences University of Rome "Foro Italico”, Piazza Lauro de Bosis 6, 00135, Rome, Italy; f Laboratory of Molecular Virology and Antimicrobial Immunity, Department of Science, Roma Tre University, 00146, Rome, Italy; g Neuroimmunology Unit, IRCCS Fondazione Santa Lucia, Via del fosso di fiorano 64, 00143, Rome, Italy; h Laboratory of Resolution of Neuroinflammation, IRCCS Fondazione Santa Lucia, Via del fosso di fiorano 64, 00143, Rome, Italy; i PhD program in Immunology, Molecular Medicine and Applied biotechnologies, University of Rome “Tor Vergata”, 00133, Rome, Italy; j Department of Systems Medicine, University of Roma "Tor Vergata", 00133, Rome, Italy; k Department of Experimental Medicine, University of Roma "La Sapienza", 00161, Rome, Italy; l Department of Biology, University of Rome “Tor Vergata”, via della ricerca scientifica, 00133, Rome, Italy; m Laboratory of Experimental Neurology, IRCCS Fondazione Santa Lucia, Via del fosso di fiorano 64, 00143, Rome, Italy

## Abstract

Amyotrophic lateral sclerosis (ALS) is a progressive neurodegenerative disease characterized by the selective loss of motor neurons. The contribution of peripheral organs remains incompletely understood. We focused our attention on brown adipose tissue (BAT) and its secreted extracellular vesicles (EVs) given their role in regulating systemic energy balance. In this study, we employed a multi-omics approach, including RNA sequencing (GEO identifier GSE273052) and proteomics (ProteomeXchange identifier PXD054147), to investigate the alterations in BAT and its EVs in the SOD1-G93A mouse model of ALS. Our results revealed consistent changes in the proteomic and transcriptomic profiles of BAT from SOD1-G93A mice, highlighting alterations such as mitochondrial dysfunction and impaired differentiation capacity. Specifically, primary brown adipocytes (PBAs) from SOD1-G93A mice exhibited differentiation impairment, respiratory defects, and alterations in mitochondrial dynamics. Furthermore, the BAT-derived EVs from SOD1-G93A mice displayed distinct changes in size distribution and cargo content. In parallel, such EVs negatively impacted the differentiation and homeostasis of C2C12 murine myoblasts, as well as induced atrophy in C2C12-derived myotubes. These findings suggest that BAT undergoes pathological perturbations in ALS mouse model and could impact on skeletal muscle homeostasis through the secretion of dysfunctional EVs.

## 1 Introduction

Amyotrophic lateral sclerosis (ALS) is a neurodegenerative disease, characterized by upper and lower motor neuron degeneration, predominantly occurring in the adult age (50–70 years of age at onset) and leading to patients’ death within 2–5 years from diagnosis [[1], [2], [3]]. ALS is characterized by progressive paralysis and atrophy of skeletal muscles, affecting also feeding behavior and the cardio-respiratory functionality. The etiology of ALS is very complex, due to the heterogeneity of the genetic background of patients with familiar ALS (approx. 10 %) as well as the high occurrence of sporadic cases (approx. 90 %) [[3], [4], [5], [6]]. The function of genes involved in ALS pathology spans from antioxidant response (SOD1), proteostasis and autophagy (C9ORF72), and RNA metabolism (TARDBP, FUS), among an increasing number of newly discovered mutations (reviewed in Ref. [7])

The main metabolic feature of ALS is the higher energy expenditure, leading to an imbalance of the energetic status. ALS patients show lower body-mass index and reduced fat mass [8,9]. Moreover, retrospective studies have shown that weight loss can occur 10 years earlier than symptoms onset and weight loss entity positively correlates with faster disease progression and worse prognosis, suggesting that the metabolic defects could have both diagnostic and prognostic values [8,10,11]. Although defective food intake could be causative for weight loss during the clinical phase of the disease [12], it does not justify the hypermetabolic state of ALS patients, as shown in indirect calorimetry assays, both in familial and sporadic cases [13,14]. Clinical biochemical characterization of ALS patients showed high incidence of hyperlipidemic status correlated with alteration of the cholesterol-lipoproteins ratios and overall defective lipid metabolism [15].

In this context, adipose tissue (AT) is the organ that systemically maintains the lipid energy homeostasis. AT accounts for the 10–30 % of tissue body weight (depending on sex) and is responsible for the storage and utilization in all mammals. Two main types of anatomically and physiologically different AT are recognized by the academic and medical community: white AT (WAT) and brown AT (BAT). White adipocytes contain one large unilocular lipid droplet that occupies the largest part of the cytoplasm, leading the nucleus to the periphery of the cell body [16]. Moreover, being mostly deputed to lipid storage, white adipocytes contain a very low number of mitochondria and are almost anabolic at rest [17]. On the contrary, brown adipocytes contain smaller lipid droplets, diffused into the cytoplasm, and a higher number of mitochondria. Mitochondria are the cellular organelle providing the real physiological function of brown adipocytes [18]. In fact, brown adipocytes display a high rate of fatty acid oxidation to maintain the highest level of mitochondria respiration and oxygen consumption. Differently from the canonical mitochondrial route, the proton-motive force is dissipated as heat by the uncoupling protein 1 (UCP1), reducing the net production of ATP by mitochondria [19]. Consistently, ATP production in BAT mostly relies on glycolysis. BAT is essential for hibernating mammals and, in human pathophysiology, correlates with healthier metabolic status and resistance to obesity and metabolic diseases since it provides removal of excess intake of dietary fatty acids (frequently occurring in western populations).

Given the metabolic role of BAT and the above reported metabolic defects in ALS patients, it is not surprising that the scientific community is considering this neglected tissue as an actor in ALS pathophysiology. Although the first reports about BAT modifications in the ALS context have been available since the 2000s, the information and data provided are often confused and contradictory.

One BAT feature that is gaining even more attention from the scientific community is extracellular vesicles (EVs) production that could mediate cell-to-cell communication. BAT is an important source of circulating EVs that can be altered, in abundance and cargo, according to physiological needs [[20], [21], [22]]. Generally, in organs with high metabolic requirements, the mitochondria homeostasis is also maintained through active secretion of damaged mitochondrial particles [23,24]. It is known that BAT and skeletal muscle share an intricate network of communication signals, including batokines, myokines and EVs [25,26]. Moreover, the BAT-EVs can alter the homeostasis of recipient cells, affecting differentiation, bioenergetics and redox state [20]. Consistently, it will be important to study the possible alterations occurring in BAT-EVs from the ALS context, which could perturb the inter-organ communication with affected districts (e.g., the skeletal muscle).

Given this premise, in this study we aim to fill the knowledge gap on the role of BAT in the pathogenesis of ALS. We performed the very-first multi-omics analysis of BAT in the SOD1-G93A mouse model at symptomatic stage, showing alteration at distinct levels, involving thermogenic program and mitochondrial homeostasis. Moreover, we characterized the phenotype of primary brown adipocytes from mouse model, showing defects in the differentiation process, bioenergetic homeostasis and lipolytic signaling. Finally, we characterized BAT-EVs with particular attention to the alteration of the EVs abundance and size distribution, as well as their bioactive role on the homeostasis of skeletal muscle cellular model.

## 2 Material and methods

### 2.1 Animal model

Transgenic hemizygous SOD1-G93A male mice (B6.Cg-Tg [SOD1 G93A]1Gur/J) were obtained from The Jackson Laboratory (Bar Harbor, ME, United States of America; RRID:IMSR_JAX:002726) and then crossbred with C57BL/6 female mice to gain offspring. Generated animals were genotyped by PCR through hSOD1 oligos using interleukin-2 (IL-2) as a PCR internal control (sequences in Supplementary Table 1).

For this study male mice of 120 days post-partum (d.p.p.) of age were employed. This stage represents the midpoint of diseased phenotype, corresponding to 25% loss of grip strength in respect to the onset time point, as previously shown in Scaricamazza et al., 2022 [27]. Mice were euthanized through cervical dislocation by professionally trained personnel.

### 2.2 RNA-preparation and RNA-sequencing

After euthanasia, mice were rinsed with 70 % ethanol and BAT was exposed, dissected with sterile scissors, and weighted. RNA was isolated with RNeasy Lipid Tissue Mini Kit (Qiagen, cat. #74804). Briefly, BAT tissue was homogenized in 1 ml of QIAzol Lysis Reagent. Phase separation was performed with addition of 200 μl of chloroform, vigorous shaking and centrifugation at 12000×*g* for 15 min at 4 °C. Aqueous phase was extracted, and 1 vol of 70 % ethanol was added to provide appropriate binding conditions. The sample was then applied to the RNeasy spin column and processed according to manufacturer's instructions. Total RNA was eluted in 30 μl of RNase-free water. For real time qPCR experiments, RNA was prepared according to standard procedures using Trizol reagent (Thermo Fisher, 15596026). RNA concentration was assessed with NanoDrop Lite Spectrophotometer. The organic phase of RNA preparation was saved apart for subsequent isolation of total proteins (see paragraph 2.3 for details). For RNA-Seq analysis, total RNA was quantified using the Qubit 4.0 fluorometric Assay (Thermo Fisher Scientific). Libraries were prepared from 125 ng of total RNA using the NEGEDIA Digital mRNA-seq research grade sequencing service (Next Generation Diagnostic srl) [28] which included library preparation, quality assessment and sequencing on a NovaSeq 6000 sequencing system using a single-end, 100 cycle strategy (Illumina Inc.). The raw data were analyzed by Next Generation Diagnostic srl proprietary NEGEDIA Digital mRNA-seq pipeline (v2.0) which involves a cleaning step by quality filtering and trimming, alignment to the reference genome and counting by gene [[29], [30], [31]]. The raw expression data were normalized, analyzed and visualized by Rosalind HyperScale architecture [32] (OnRamp BioInformatics, Inc.). Data are available at GEO identifier GSE273052.

### 2.3 Total protein preparation and mass spectrometry analysis

The organic phase from RNA preparation (see paragraph 2.2 for details) was processed for total protein preparation, according to manufacturer's instructions. Briefly, the interphase was removed and 0.3 ml of ethanol 100 % were added, incubated at RT and centrifuged at 2000×*g* for 5 min at 4 °C. The phenol-ethanol supernatant was transferred to a new tube and 1.5 ml isopropanol were added, incubated for 10 min at RT and centrifuged at 12000×*g* for 10 min at 4 °C to pellet proteins. Protein pellets were washed four time with 2 ml of 0.3 M guanidinium hydrochloride solution in 95 % ethanol, with 20 min of incubation at RT and centrifugation at 7500×*g* for 5 min at 4 °C. The last wash was performed with 2 ml of 100 % ethanol and the pellet was air-dried for 10 min. The pellet was resuspended in 0.2 ml of SDS 1 % and heated for 15 min at 50 °C to allow complete dissolution. Protein concentration was determined with Bradford assay. Protein leftover was processed for western blotting as described above. Instruments for LC-MS/MS analysis consisted of a NanoLC 1200 coupled via a nano-electrospray ionization source to the quadrupole-based Q Exactive HF benchtop mass spectrometer. Peptide separation was carried out according to their hydrophobicity on a PicoFrit column, 75 μm ID, 8 μm tip, 250 mm bed packed with Reprosil-PUR, C18-AQ, 1.9 μm particle size, 120 Å pore size (New Objective, Inc., cat. PF7508-250H363), using a binary buffer system consisting of solution A: 0.1 % formic acid and B: 80 % acetonitrile, 0.1 % formic acid. Total flow rate: 300 nl/min. After sample loading, run start at 5 % buffer B for 5 min, followed by a series of linear gradients, from 5 % to 30 % B in 90 min, then a 10 min step to reach 50 % and a 5 min step to reach 95 %. This last step was maintained for 10 min. MS spectra were acquired using 3E6 as an AGC target, a maximal injection time of 20 ms and a 120,000 resolution at 200 *m*/*z*. The mass spectrometer operated in a data dependent Top20 mode with subsequent acquisition of higher-energy collisional dissociation (HCD) fragmentation MS/MS spectra of the top 20 most intense peaks. Resolution for MS/MS spectra was set to 15,000 at 200 *m*/*z*, AGC target to 1E5, max injection time to 20 ms and the isolation window to 1.6 Th. The intensity threshold was set at 2.0E4 and Dynamic exclusion at 30 s. All acquired raw files were processed using MaxQuant (1.6.2.10) and the implemented Andromeda search engine. For protein assignment, spectra were correlated with the *Mus musculus* (v. 2021) including a list of common contaminants. Searches were performed with tryptic specifications and default settings for mass tolerances for MS and MS/MS spectra. The other parameters were set as follow.

• Fixed modifications: Propionamide(C)
• Variable modifications: Oxidation, Acetyl (N-term).
• Digestion: Trypsin
• Min. peptide length = 7
• Max. peptide mass = 470 Da
• False discovery rate for proteins and peptide-spectrum = 1 %

For further analysis, the Perseus software (1.6.2.3) was used and first filtered for contaminants and reverse entries as well as proteins that were only identified by a modified peptide [First filter]. The LFQ Ratios were log-transformed, grouped and filtered for min.valid number (min. 3 in at least one group) [Second filter]. Missing values have been replaced by random numbers that are drawn from a normal distribution. A two-sample *t*-test analysis was performed with FDR = 0.05. Proteins with Log2 Difference ≥ ± 1 and q-value <0.05 were considered significantly enriched. Data is available at ProteomeXchange identifier PXD054147.

### 2.4 Isolation and characterization of BAT-extracellular vesicles

Extracellular vesicles was isolated as previously described [20]. Briefly, BAT was dissected, washed with sterile PBS and minced into small pieces in 0.5 ml of isolation medium (DMEM/F12, 1 % Pen/Strep) and incubated in standard conditions for 16h. Supernatants were filtered through 30 μm nylon mesh and centrifuged at 600×*g* for 10 min at 4 °C to remove debris. The resulting suspension was ultracentrifuged at 100000×*g* for 2h at 4 °C in a swinging bucket rotor. For proteomics and western blotting, EVs pellets were resuspended in RIPA buffer and processed as described above. To assess both the concentration and size of the isolated vesicles, Nanoparticle-tracking analysis (NTA) was performed. All samples were quantified using a Nanosight NS300 (Malvern Panalytical, UK) equipped with a 532 nm laser. The acquisition was made using a camera level of 15 and involved capturing five 60-s videos for each sample, followed by analysis. The pellets were resuspended in pre-filtered PBS, and according to the manufacturer's instructions, samples were appropriately diluted (range: 1:50 to 1:200) to achieve a reading-compatible concentration.

### 2.5 Integrated functional enrichment analysis of proteomics and RNA-seq data

Upregulated genes from RNA-Seq and proteins from BAT-tissue proteomics were integrated into the Metascape online platform [33]. Same procedure was performed for the integration of upregulated proteins from BAT-tissue and BAT-EVs proteomics. The resulting data were retrieved from the software and used for results description without further manipulation.

### 2.6 Isolation, culture, and treatment of brown primary pre-adipocytes

Isolation and culture of mouse primary brown pre-adipocytes (mPBAs) was performed according to Ngo et al., 2021 [34] with minor modifications. BAT was washed with sterile ice-cold PBS and minced into small pieces into 0.5 ml of collagenase solution (HBSS w/Ca^2+^/Mg^2+^/glucose, 3.5 % BSA, 1 % Pen/Strep, Amphotericin B 500 ng/ml, Gentamycin 40 μg/ml). Tissue pieces were transferred into a sterile 15 ml tube containing 6.5 ml of the same solution (total 7 ml). Collagenase type I (Gibco, cat. # 17100017) and type II (Gibco, Cat # 17101015) were added at final concentration of 1 mg/ml. Tubes were placed under gentle agitation for 1h at 37 °C, with vortexing every 15 min. Tissue homogenates were filtered with 100 μm nylon mesh and centrifuged at 250×*g* for 5 min at RT, inverted 10 times and centrifuged again. Supernatants were discarded and cell pellets were treated with 1 ml of red blood cell lysis buffer (NH_4_Cl 154 mM M, K_2_HPO_4_ 10 mM, EDTA 0.1 mM), incubated for 5 min at RT, resuspended into 13 ml of washing buffer (HBSS w/o Ca^2+^/Mg^2+^ w/glucose, 3.5 % BSA, 1 % Pen/Strep, Amphotericin B 500 ng/ml, Gentamycin 40 μg/ml) and filtered through a 40 μm nylon mesh. Cells were centrifuged at 500×*g* for 5 min at RT and resuspended in amplification medium (Cytogrow, RESNOVA, Cat. # TGM-9001-B) and counted. 2 × 10^5^ cells were seeded on 100 mm Ø dish and cultured for 72–96h in standard conditions. Cultures were upscaled or downscaled according to the number of cells isolated. After amplification, mPBAs were seeded for appropriate experiment in growth medium GM (DMEM/F12, 10 % FBS, 1 % Pen/Strep) at density of 1.4x10^5^ cells/cm^2^. After 3 days in GM, induction of brown adipogenic differentiation was performed for 48 h in adipogenic induction medium AIM (GM + dexamethasone 1 μM, 3-isobutyl-methyl xanthine IBMX 0.5 mM, indomethacin 0.125 mM, rosiglitazone 1 μM, T3 1 nM and insulin 1 μg/ml). After the induction, differentiation was continued in adipogenic maintenance medium AMM (GM + rosiglitazone 1 μM, T3 1 nM and insulin 1 μg/ml) until day 8, with medium change every 2 days. For isoproterenol treatment, cells were stimulated for 16h with 1 μM isoproterenol.

### 2.7 Culture and treatment of C2C12 murine myoblasts

C2C12 murine myoblasts were purchased from ATCC (Ref. C2C12 CRL-1772™) and cultured in DMEM high glucose, supplemented with 10 % FBS, 1 mM sodium pyruvate, 2 mM glutamine and 1 % Pen/Strep. For differentiation, cells were seeded at 1 × 10^4^ cells/cm^2^ and allowed to reach confluence for 72 h. Differentiation was induced with differentiation medium, consisting of DMEM high glucose, supplemented with 2 % horse serum, 1 mM sodium pyruvate, 2 mM glutamine and 1 % Pen/Strep. For treatments with BAT-EVs, vesicles were resuspended in sterile 1 × PBS. Protein concentration was assessed with Bradford assay. Then, EVs were aliquoted and stored at −80. For growth curve experiment, cells were seeded at 5 × 10^3^ cells/cm^2^ and treated after over-night incubation with increasing concentration of BAT-EVs (0-0.5-1-2-4-8-16 μg/ml) for four time points (0-24-48-72 h). For viability MTS experiment, cells were seeded at 4 × 10^4^ cells/cm^2^ and treated after over-night incubation with increasing concentration of BAT-EVs (0-0.5-1-2-4-8-16 μg/ml). for differentiation assay, cells were treated with 3.5 μg/ml of BAT-EVs and allowed to differentiate for 24 and 48 h (for Western blot) or up to 72 h (for fusion index analysis). For the electron flow assay, cells were seeded at 4 × 10^4^ cells/cm^2^ and treated after over-night incubation with 3.5 μg/ml of BAT-EVs and analyzed after 24 h. For atrophy experiments, myotubes were treated with 3.5 μg/ml of BAT-EVs and analyzed after 24 h.

### 2.8 Cell lysis, protein extracts and western blotting analysis

Cells were washed 2 times with ice cold PBS and lysed in appropriate volume of RIPA buffer [Tris base 50 mM pH 8.0, 1 % Triton X-100, 0.25 % sodium deoxycholate, 0.1 % SDS, NaCl 250 mM, 1 mM EDTA, 5 mM MgCl_2_, 1 mM sodium orthovanadate, 1 mM NaF, 1 mM phenylmethylsulfonyl fluoride, 1X Protease Inhibitor Cocktail (Sigma-Aldrich, #P8340), 1X Phosphatase Inhibitor Cocktail 2 (Sigma-Aldrich, #P5726), 1X Phosphatase Inhibitor Cocktail 3 (Sigma-Aldrich, #P0044)] and incubated in ice for 30 min. Lysates were sonicated using a probe sonicator and then centrifuged at 15,500×*g* at 4 °C for 30 min. Protein quantitation was assessed with Bradford Bio-Rad Protein Assay Dye Reagent Concentrate (Bio-Rad, #5000006). Protein extracts were treated with 4X Laemmli buffer (0.25 M Tris base pH 6.8, 8 % SDS, 40 % glycerol, 20 % 2-mercapto-ethanol, 4 mg/ml bromophenol blue), and heated at 96 °C for 10 min, then stored at −20 °C until used. 5–20 μg of proteins were loaded onto 4–20 % SDS-polyacrylamide gel and run with tris-glycine-SDS buffer (0.025 M tris base, 0.192 M glycine, 0.01 % SDS, pH 8.3). Gels were blotted onto nitrocellulose membranes (0.22 μm pore size) at 30V for 16h at 4 °C in tris-glycine-ethanol buffer (0.025 M tris base, 0.192 M glycine, pH 8.3; 20 % ethanol). Membranes were saturated with blocking buffer consisting of 5 % non-fat dried milk in TBS-Tween buffer (0.02 M tris base, 0.15 M NaCl, 0.001 Tween-20). Primary antibodies were diluted appropriately in blocking buffer and incubated for 16h at 4°c under gentle agitation. Secondary antibodies were appropriately diluted in blocking buffer and incubated for 1h at RT under gentle agitation. Chemiluminescence was detected with Clarity™ Western ECL Substrate (Biorad) through iBright Imaging System (Thermo Fisher Scientific). Complete list of primary and secondary antibodies and dilutions is available in Supplementary Table 2. Densitometric analysis was performed with Fiji software [35].

### 2.9 cDNA synthesis and real time qPCR

cDNA synthesis was performed with ImProm-II™ Reverse Transcription System (Promega, cat. # A3800) according to manufacturer's instructions. Real time qPCR analysis was performed with LightCycler 480 SYBR Green System (Roche, ETC). We used the “second derivative max” algorithm of the LightCycler software to calculate the crossing point (Cp) values. TATA box-binding protein as the housekeeping gene for normalization. Primer sequences are listed in Supplementary Table 3.

### 2.10 Adipocyte staining and analysis

Adipocyte staining was performed with Oil Red-O (ORO) as previously described [[36], [37], [38], [39]]. ORO stock solution was prepared at 3 mg/ml in iso-propanol, stirred over night at 4 °C and filtered with standard filter paper. Cells were fixed with 4 % paraformaldehyde solution, for 10 min at RT, washed 3 times with PBS, 1 time with ultrapure water and 1 time with 60 % iso-propanol. Adipocytes were stained with appropriate volume of ORO working solution (OROstock:water 3:2, 0.22 μm filtered) for 20 min at RT under gentle agitation. After staining, nuclei were counterstained with DAPI following standard procedure. Images were acquired with ZOE Fluorescent Cell Imager (Biorad). ORO positive area and nuclei number was analyzed with a dedicate pipeline in Cell Profile software [40]. In particular, fluorescent images were acquired from four different cell preparations from independent mice (n = 4). The pipeline identifies the ORO-positive area and the nuclei. Image mask was used to relate each nucleus with its respective neighboring ORO-positive area, thus identifying each adipocyte. Adipocytes' diameter was estimated and expressed as μm^2^. In total, 3845 wild type and 4021 SOD1-G93A adipocytes were identified and cumulated. The normality of distribution was assessed through the D'Agostino & Pearson test. Statistical significance was assessed with the Kolmogorov-Smirnoff test for non-normal distribution.

### 2.11 Immunofluorescence assay, fusion index analysis and myotube diameter

Cells were fixed with 4 % PFA in PBS, for 10 min at RT and then washed with PBS. Permeabilization was performed with 0.5 % triton-X 100 in PBS for 5 min. Blocking was performed with 10 % FBS and 0.1 % Triton-X 100 in PBS for 1 h under gentle agitation (5 rpm). Primary antibody anti myosin heavy chain (MF-20, DSHB) was diluted at 2 μg/ml in blocking solution and incubated for 2 h at RT under gentle agitation (5 rpm). After 3 washes with 0.1 % Triton-X 100 in PBS, secondary antibody (Goat anti-mouse IgG (H + L) cross-adsorbed secondary antibody, Alexa Fluor 488, Thermo Fisher Scientific, Cat. #A-11001) was diluted 1:500 in blocking solution and incubated for 1 h at RT under gentle agitation. After 3 washing steps, nuclei were counterstained with Hoechst 33342 (Invitrogen, Cat. no. H3570) diluted 1:5000 in PBS. Images were acquired with ZOE Fluorescent Cell Imager (Biorad) for analysis and with LSM 800 Confocal Laser Scanning Microscope (Zeiss) for representative images. Fusion index analysis was performed with a dedicated pipeline in Cell Profile Software [40]. Briefly, the pipeline first recognizes total nuclei in the Hoechst channel. Then, the MHC-positive area is detected and masked over the image of nuclei. Myonuclei are counted in the masked image using the same parameters of total nuclei. Fusion index is expressed as percentage of myonuclei over total nuclei. Myotube diameter analysis was performed with the Image J macro “Myotube width macro v.62” [41], with minor modification. Briefly, the pipeline takes the MHC-positive channel and skeletonizes the area of each myotube. Then, the average angle of all the myotubes is calculated and the software draws a line perpendicular to the average angle. The intersection between the line and the myotubes is used to measure the myotube diameter, expressed in micrometers.

### 2.12 Mitochondrial morphological analysis

Primary brown pre-adipocytes were seeded at density of 1.4x10^4^ cells/cm^2^ (1/10 in respect to the differentiation protocol) and fixed with 4 % PFA after overnight incubation in standard culture condition. The mitochondrial network was labelled with a standard immunofluorescence protocol with anti-TOMM20 antibody (Supplementary Table 2) and goat anti-mouse Alexa fluor 488 secondary antibody (Thermo Fisher Scientific, cat #A-11008). Images were acquired with Olympus BX51 Microscope. Mitochondrial network analysis was performed with the MiNA Plugin in the Image J software according to the standard analysis workflow indicated in the publication [42].

### 2.13 Bioenergetic analysis

Bioenergetic analysis was performed with the Seahorse XFe96 instrument (Agilent Technologies). mPBAs were seeded into Seahorse XF microplates (Agilent Technologies, Cat. #103794-100) at 15000 cells/well density in GM and cultured for 3 days in standard conditions. Differentiation protocol was performed as described above. Mitochondria-stress test assay was performed in Seahorse XF DMEM pH 7.4 medium (Agilent Technologies, Cat. #103575-100) supplemented with 1 mM sodium pyruvate, 10 mM D-glucose and 2 mM glutamine. Mitochondria were stressed with 1 μM oligomycin A, 1.5 μM FCCP and 1 μM rotenone/antimycin. Data were analyzed in the Seahorse Analyzer web platform (Agilent Technologies).

Electron flow assay on C2C12 myoblasts was performed in mitochondria assay solution (MAS), consisting of 220 mM mannitol, 70 mM sucrose, 10 mM KH_2_PO_4_, 5 mM MgCl_2_, 2 mM HEPES, 1 mM EGTA, 0.2 % fatty acid-free BSA (Sigma Aldrich, cat. #A7030), 10 mM pyruvate, 1 mM malate, 4 mM ADP and 1 nM plasma membrane permeabilizer (Agilent Technologies, Cat #102504-100). Cells were incubated for 20 min in MAS, at 37 °C in ambient air and analyzed immediately after. Complex I was inhibited with 2 μM rotenone, Complex II was stimulated with 10 mM succinate while Complex III was inhibited with 2 μM antimycin. Equilibration step was skipped. Measurements were performed 3 times with mix/wait/measures times of 30 s/30 s/2 min, for each injection step.

Raw data were normalized per cell number. To remove the batch effect of different Seahorse experiments, data were normalized versus the mean baseline value of wild type samples in each plate.

### 2.14 Mitochondrial potential and flow cytometry

Mitochondrial potential was measured by flow cytometry. Briefly, cells were washed in PBS and stained with MitoTracker Red (Molecular Probes, Eugene, OR) for 30 min in culture medium in standard culture conditions, at a final concentration of 500 nM according to manufacturer's instructions. Mitochondrial fluorescence was analyzed with a CytoFLEX flow cytometer (Beckman Coulter). Excitation was achieved using the 640 nm laser. Gating parameters on the flow cytometer were established using a control sample and adjusting the voltages for the forward scatter, side scatter and MitoTracker Red laser. Data were analyzed by FlowJo software (Treestar, Ashland, OR, USA).

### 2.15 Statistical analysis

Statistical analysis for wet-lab experiments was performed in GraphPad Prism Software v10. Normality of datasets was assessed by D'Agostino-Pearson Test. Outliers were removed with ROUT method with Q = 1 %. Hetero/Homoscedasticity was assessed by the F test to compare variances. Comparison between two groups was performed with T-test, with appropriate correction for normality/non-normality and hetero/homoscedasticity as indicated in the relative figure legend. Comparison between more than two groups was performed by One-of Two-Way ANOVA test with multiple comparison correction when appropriate. For all comparisons, significant results were taken when p value < 0.05.

## 3 Results

### 3.1 Multi-omics analysis of brown adipose tissue reveals alterations in an ALS-mouse model

**Fig. 1:**
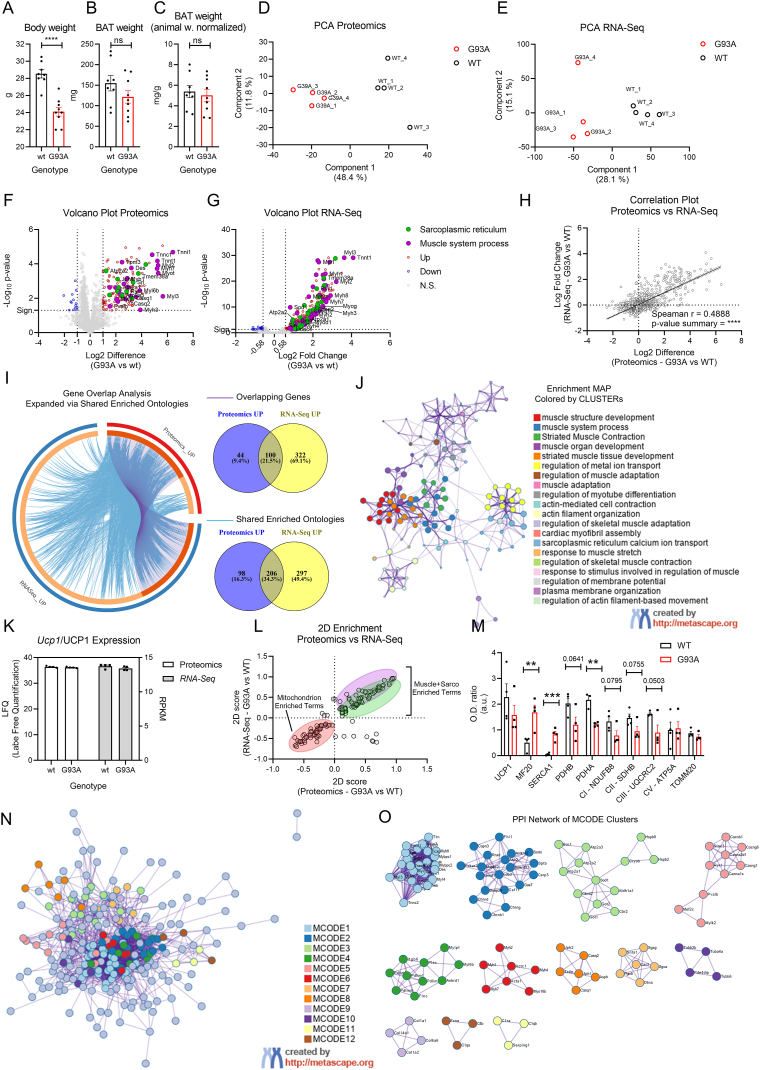
Characterization of BAT from wild type and G93A mouse model A-B-C) Bar plot representing mouse model weight, BAT raw and normalized weight. D-E) dispersion plot representing the principal component analysis of proteomics and RNA-Seq data. F-G) volcano plot of the proteomics and RNA-Seq data. H) dispersion plot representing the correlation analysis between proteomics and RNA-Seq datasets. I) chord diagram and Venn diagrams representing the genes and ontology overlap between proteomics and RNA-Seq up-regulated entities. J) enrichment map of commonly enriched ontology terms in proteomics and RNA-Seq up-regulated entities. Color coding identifies the different clusters of enriched terms. K) bar plot representing the raw quantification data of the UCP1/Ucp1 protein/gene in proteomics and RNA-Seq datasets, respectively. L) dispersion plot of the 2D enrichment analysis between proteomics and RNA-Seq data of BAT. M) bar plot representing the OD ration form densitometric analysis of Western blot shown in Fig. S2 D-F. Significance was assessed by Student's t-test. ∗∗p < 0.01; ∗∗∗p < 0.001. N) PPI network colored by MCODE clusters, relative to the Metascape integrated analysis of proteomics and RNA-Seq. O) PPI network of the separated MCODE clusters from panel N. Entities are reported as gene names.

Since the information about the role and the eventual modifications occurring at the level of BAT in the ALS contexts are often fragmented and contradictory, we decided to fill this gap with a complete characterization of BAT at the symptomatic stage of the disease, by a multi-omics approach involving both proteomics (ProteomeXchange identifier PXD054147) and RNA-Sequencing (GEO identifier GSE273052) analyses on BAT total extracts from wild type and SOD1-G93A mouse model at symptomatic stage of 120 d.p.p. As expected, SOD1-G93A mice show a reduction in body weight, confirming that atrophic process, characteristic of this ALS model, has been through (Fig. 1A). Nevertheless, BAT did not show significant reduction in tissue mass neither at net weight nor normalized per body weight, suggesting that this tissue does not undergo excessive exhaustion at this stage (Fig. 1B–C).

To unveil molecular features characterizing the BAT from SOD1-G93A mice, we performed proteomics and RNA-Seq on total protein and RNA preparations. Correlation matrix showed high intragenotype similarity with proteomics Pearson coefficient 0.95 < r < 0.97 and RNA-Seq Pearson coefficient 0.97 < r < 0.98 (Supp. Figs. 1A–B). Principal component analysis (PCA) showed a sharp intergenotype segregation mainly across component 1, accounting for 48.4 % and 28.1 % in proteomics and RNA-Seq experiments, respectively (Fig. 1D–E). Differential expression analysis was performed separately for both datasets in the Perseus environment for proteomics [43] and in the Rosalind environment for RNA-Seq [32]. Mass spectrometry allowed the identification of 3377 proteins, resulting in 2364 entities after filtering process. Among the 158 differentially expressed proteins (DEPs), 144 were overrepresented while 14 were underrepresented, considering q-value <0.05 and |Log2 Difference| > 1 (Fig. 1F and Supp. Fig. 1C). RNA-Seq analysis allowed the identification of 437 differentially expressed genes (DEGs), consisting of 422 upregulated and 15 downregulated DEGs, considering adj. p-value <0.05 and |fold change| > 1.5 (Fig. 1G e Supp. Fig. 1D). Since the two datasets showed positive correlation (Fig. 1H), enrichment and ontology analyses were performed in the Metascape on-line platform [33], considering the integration of overrepresented/upregulated entities from both datasets. The Gene Overlap Analysis Expanded via Shared Enriched Ontologies showed that the two datasets share 100 common entities and 206 enriched ontologies (Fig. 1I). Enriched ontology cluster network showed significant enrichment of terms mainly related to two macro-categories: acto-myosin complex and sarcoplasmic reticulum/calcium homeostasis (Fig. 1J, Supp. Figs. 2A–C). Interestingly, entities belonging to these categories are the ones driving the intergenotype segregation both in volcano-plots and in PCA analyses with particular focus on sarcolipin (*Sln*) gene being the predominant driver gene over component 1 in the RNA-Seq experiment (Supp. Figs. 1E–F). Among the mostly dysregulated entities, increased expression of myosin heavy/light chain isoforms and SERCA pumps predominantly characterized BAT from SOD1-G93A mouse model (Supp. Figs. 1G–H) suggesting that this tissue undergoes a molecular rearrangement imputable to the activation of alternative/non-canonical thermogenic mechanisms. This hypothesis is corroborated by the absence of modulation of Ucp1/UCP1 gene/protein expression in SOD1-G93A BAT (Fig. 1K–L). To deeper investigate the overall alterations in the molecular environment of BAT in the ALS context, we performed 2D-Enrichment analysis between total proteome and transcriptome in the Perseus platform [43]. Results confirmed the positive enrichment of Actomyosin- and Sarcoplasmic Reticulum-related terms, while also showing depletion of Mitochondria-related ones (Fig. 1L and Supp. Fig. 1I). Although mitochondria genes and proteins did not result from the previous statistical analyses, this information was confirmed by 2D Fisher test with Benjiamini-Hoechberg FDR cut-off <0.02. Western blot analysis on total protein extracts from BAT confirmed the increase in abundance of sarcomeric myosin heavy chain (MHC) and SERCA1 ATPase (Fig. 1M, Supp. Fig. 1D) and corroborates the information related to decreased abundance of mitochondrial proteins, relatively to the inner matrix metabolic enzymes, pyruvate dehydrogenase subunit alpha (PDHA), pyruvate dehydrogenase subunit beta (PDHB), pyruvate carboxylase beta (PCB) (Fig. 1M, Supp. Fig. 1E) and electron transport chain complexes (Fig. 1M, Supp. Fig. 1F).

To gain further insights into the dynamics occurring at the molecular level between the dysregulated entities resulted from proteomics and RNA-Seq analyses, we adopted a network approach. Protein-protein interaction network analysis showed high interconnection between entities from both proteomics and RNA-Seq (Supp. Fig. 2G). MCODE clustering analysis recognized 12 different clusters in the PPI network (Fig. 1N–O, Table 1). MCODE1, MCODE2, MCODE4, MCODE6 and MCODE7 represented ontology terms related to the acto-myosin complex and muscle-related terms suggesting the activation of alternative thermogenic program through myosin-dependent futile ATP consumption. MCODE 3, MCODE5, and MCODE8 showed enrichment for pathways related to the intracellular calcium homeostasis, consisting in overexpression of SERCA pumps (Atp2a1, Atp2a2, Atp2a3), mitochondrial casein kinase (Ckmt2), mitochondrial and cytosolic transamination (Got1, Got2) and antioxidant response to lipid peroxidation (Aldh1a1), calcium voltage-gated channels (CACNs), ryanodine receptor (Ryr1), parvalbumin (Pvalb) and calsequestrins (Casq1, Casq2). These data suggest that calcium homeostasis is perturbed in BAT from SOD1-G93A mouse model and could be pivotal for the activation of ATP-consuming pathways contributing to alternative thermogenesis. Interestingly, MCODE9, MCODE10, MCODE11 and MCODE 12 report enrichment for other altered pathways such as collagen deposition, intracellular transport and the complement cascade, which could suggest dysregulation in parallel cellular functions that need further investigation.

**Table 1:** MCODE clustering details.

Network	Annotation		
	Term ID	Term Description	p-value (-Log10)
MCODE1	R-MMU-390522	Striated Muscle Contraction	−69.5
	R-MMU-397014	Muscle contraction	−48.7
	GO:0006936	muscle contraction	−48.6
MCODE2	GO:0061061	muscle structure development	−11
	GO:0042692	muscle cell differentiation	−8
	GO:0030036	actin cytoskeleton organization	−6.6
MCODE3	R-MMU-418359	Reduction of cytosolic Ca++ levels	−7.2
	GO:0090075	relaxation of muscle	−6.7
	R-MMU-418360	Platelet calcium homeostasis	−6.3
MCODE4	GO:0061061	muscle structure development	−13.2
	GO:0030029	actin filament-based process	−8.9
	GO:0030036	actin cytoskeleton organization	−7.3
MCODE5	mmu04921	Oxytocin signaling pathway - *Mus musculus* (house mouse)	−15.6
	GO:1902514	regulation of calcium ion transmembrane transport via high voltage-gated calcium channel	−10.1
	GO:0070588	calcium ion transmembrane transport	−10
MCODE6	mmu04814	Motor proteins - *Mus musculus* (house mouse)	−14.3
	GO:0006936	muscle contraction	−8.8
	GO:0003012	muscle system process	−8.2
MCODE7	CORUM:349	Sarcoglycan-sarcospan-syntrophin-dystrobrevin complex	−12.9
	mmu05412	Arrhythmogenic right ventricular cardiomyopathy - *Mus musculus* (house mouse)	−11.3
	mmu05416	Viral myocarditis - *Mus musculus* (house mouse)	−11
MCODE8	GO:1901019	regulation of calcium ion transmembrane transporter activity	−11.8
	GO:0051279	regulation of release of sequestered calcium ion into cytosol	−11.4
	GO:2001257	regulation of cation channel activity	−10.8
MCODE9	R-MMU-8948216	Collagen chain trimerization	−11.2
	R-MMU-2022090	Assembly of collagen fibrils and other multimeric structures	−10.7
	R-MMU-1650814	Collagen biosynthesis and modifying enzymes	−10.2
MCODE10	R-MMU-190840	Microtubule-dependent trafficking of connexons from Golgi to the plasma membrane	−8.8
	R-MMU-190872	Transport of connexons to the plasma membrane	−8.8
	R-MMU-9668328	Sealing of the nuclear envelope (NE) by ESCRT-III	−8.2
MCODE11	R-MMU-977606	Regulation of Complement cascade	−8.3
	GO:0006958	complement activation, classical pathway	−8.3
	GO:0002455	humoral immune response mediated by circulating immunoglobulin	−8.1
MCODE12	R-MMU-166663	Initial triggering of complement	−9.1
	R-MMU-166658	Complement cascade	−8
	GO:0006956	complement activation	−7.8

Table reporting the list of MCODE cluster recognized by the integrated Metascape analysis of proteomics and RNA-Seq data showed in Fig. 1N–O. For each cluster, the top 3 enriched terms are reported, relatively to the lowest p-value.

### 3.2 Primary brown adipocytes from SOD1-G93A mice display differentiation impairment and respiratory defects

**Fig. 2:**
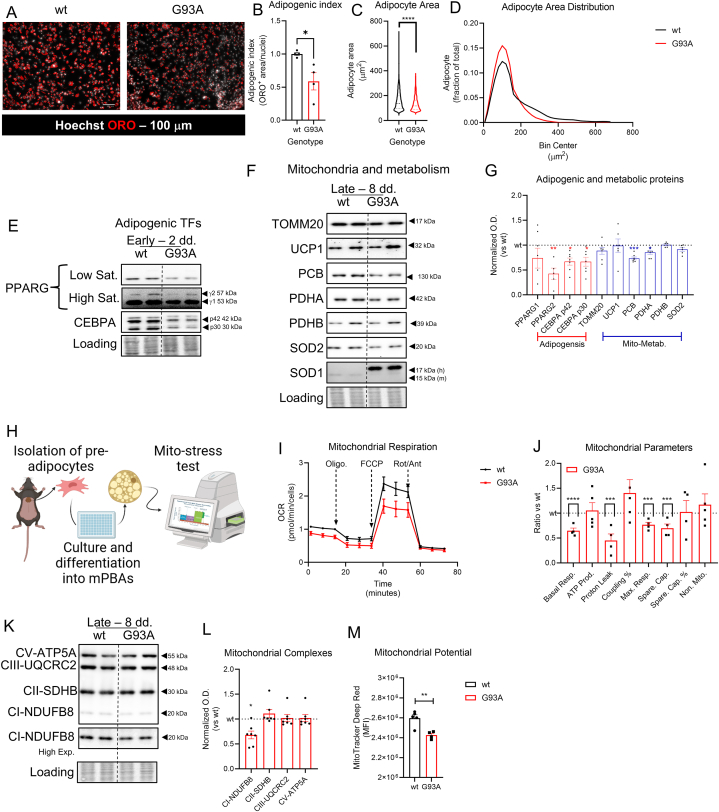
Characterization of mPBAs from SOD1-G93A mouse model. A) representative fluorescent micrographs of mPBAs from wild type and SOD1-G93A mouse models. Lipid content was labelled with Oil-red O (ORO) and nuclei were counterstained with Hoechst 33342. Scale bar = 100 μm. B) bar plot representing the quantitation of the adipogenic index relative to experiment in panel A. Data were obtained as ORO-positive area normalized per nuclei and expressed as mean ± SEM. Statistical significance was assessed through Welch's test. ∗p < 0.05. n = 4 C) violin plot representing the quantitation of adipocyte area from images panel A. Data were obtained through automatic image analysis and expressed as μm^2^. Dashed line represents the median value. Dotted lines represent the 1st and the 3rd quartiles. Statistical significance was assessed through the Kolmogorov-Smirnoff test. See paragraph 2.10 for analysis details. ∗∗∗∗p < 0.0001. D) distribution plot of adipocytes area from images in panel A. Data were obtained as for panel C and represented as frequency distribution. E) representative Western blot analysis of adipogenic transcription factors PPARG and CEBPA analyzed at 48 h after adipogenic induction. PPARG is shown at low and high saturation level to allow the appreciation of both the bands of isoform 1 and 2. Ponceau red staining was used as loading control. F) representative Western blot of late mitochondrial and metabolic markers TOMM20, UCP1, PCB, PDHA, PDHB, SOD2 analyzed at 8 days of differentiation. SOD1 antibody was used as internal control for human SOD1 overexpression in SOD1-G93A mouse model. Ponceau red staining was used as loading control. G) bar plot representing the densitometric quantitation of Western blot experiments in panels E–F. Data were obtained by normalization versus loading control and expressed as ration versus the mean of wild type samples. Statistical significance was assessed through Student's t-test. ∗p < 0.05; ∗∗p < 0.01; ∗∗∗∗p < 0.0001. n_wt_ = 4; n_G93A_ = 7. H) cartoon representing the experimental model for Seahorse experiment. Pre-adipocytes were isolated from BAT and expanded in Cytogrow. Thereafter, cells were seeded onto Seahorse microplates and let to differentiate into mPBAs for 8 days. Mito-stress test was conducted with Oligomycin 1 μM, FCCP 1.5 μM and Rotenone/Antimycin A 1/1 μM. Artwork was mad with Biorender www.biorender.com I) graph representing the oxygen consumption rate (OCR) of mPBAs from wild type and SOD1-G93A mice during the mito-stress test, as described in panel H. Data are reported as time on the x axis, measured in minutes, and OCR on the y axis, normalized versus the baseline value of wild type samples. J) bar-plot representing the mitochondrial respiratory parameters from Seahorse experiments. Data were obtained through the Seahorse analytics platform www.seahorseanalytics.agilent.com. Data are reported as mean ± SEM and normalized versus the mean of wild type samples. Statistical significance was assessed through the Student's t-test. ∗∗∗p < 0.001; ∗∗∗∗p < 0.0001. n = 5. K) representative Western blot of mitochondrial ETC complexes NDUFB8, SDHB, UQCRC2 and ATP5A. NDUFB8 is shown also at higher exposure time. L) bar plot representing the densitometric quantitation of Western blot in panel K. Data are expressed as mean ± SEM and normalized versus the mean value of wild type samples. Statistical significance was assessed through Student's t-test. ∗p < 0.05 Wild type is shown as reference dotted line at y = 1. n_wt_ = 4; n_G93A_ = 7. M) bar plot representing the quantitation of mitochondrial transmembrane potential through MitoTracker deep red staining in flow-cytometry. Data are reported as mean fluorescent intensity ± SEM. Statistical significance was assessed through Student's t-test. ∗∗p < 0.01. n = 5.

Multi-omics analysis highlighted several alterations in BAT from symptomatic SOD1-G93A mouse model, mainly consisting of activation of alternative thermogenic pathways and impairment of the mitochondrial compartment. To better characterize such defects, we performed experiments aimed at analyzing the phenotypical demeanor and the metabolic functionality of *ex vivo* cultured murine primary brown adipocytes (mPBAs). mPBAs from SOD1-G93A mice showed reduced adipogenic index, expressed as Oil Red-O positive area normalized per total number of cells in fluorescence microscopy imaging (Fig. 2A–B). Moreover, morphological screening of adipocytes through automated image analysis showed reduced adipocyte area (Fig. 2C–D). In particular, mPBAs showed reduced differentiation and almost absence of adipocytes with area greater than 400 μm^2^ (Fig. 2 D). To obtain further information on the molecular signaling controlling adipogenic differentiation we performed Western blot analysis throughout the differentiation process. After 48 h from induction of differentiation with AIM medium (see paragraph 2.6 for composition details), the transcriptional cascade controlled by PPAR γ1/γ2 and CEBPα was downregulated (Fig. 2E–G). In particular, PPAR γ2 showed the lowest expression, compared to the γ1 isoform, suggesting a differential regulation between lipogenesis and mitochondrial metabolism. At terminal differentiation, after other 6 days in AMM medium (see paragraph 2.6 for composition details), we dissected different aspects of the adipogenic differentiation, including mitochondria and metabolic enzymes. As showed in the proteomic and transcriptomic profiling, UCP1 protein levels do not change consistently in SOD1-G93A mPBAs (Fig. 2F–G). Similarly, mitochondrial mass marker TOMM20 remains stable, suggesting that variation in mitochondrial proteins and genes highlighted by the multi-omics characterization does not involve the total mitochondrial network, but relies on the downregulation of mitochondrial metabolic proteins, as for the case of PDHA, PDHB and PCB. Moreover, antioxidant response, mediated by SOD2 protein does not seem to be activated, suggesting absence of increased redox flux in SOD1-G93A mPBAs (Fig. 2F–G). Overall, phenotypical characterization clearly shows adipogenic and metabolic defects in mPBAs from SOD1-G93A mouse model. Since multi-omics analysis and Western blot showed alteration at the mitochondrial compartment, we next performed Extracellular Flux analysis through Seahorse Technology™ in order to assess the mitochondrial functionality (Fig. 2H). Results show a drastic reduction in mitochondrial respiration (Fig. 2 I), with particular modulation of Basal and Maximal Respiration, Spare Capacity and Proton Leak (Fig. 2 J), confirming an impairment of mitochondrial functionality (Fig. 1L–M). Modulation in Basal and Maximal Respiration prompted us to assess the abundance of mitochondrial ETC complex by Western blot. Interestingly, Complex I subunit NDUFB8 is downregulated in SOD1-G93A mPBAs (Fig. 2K–L). This result could be correlated with respiration alterations, as Complex I downregulation could be responsible for a lower reducing-equivalent flux through the electron transport chain. This could be at the basis of also lower Spare Capacity and Proton Leak, as confirmed by the analysis of MitoTracker Red staining by flow-cytometry, showing reduced mitochondrial potential (Fig. 2 M). Overall, the results highlight that mPBAs from SOD1-G93A mice have differentiation impairment, probably due also to a lower mitochondrial performance unable to support a proper adipogenic differentiation. These defects could also affect cellular response to external stimuli.

### 3.3 mPBAs from SOD1-G93A mice have alterations of mitochondrial dynamics and response to pro-lipolytic stimuli

**Fig. 3:**
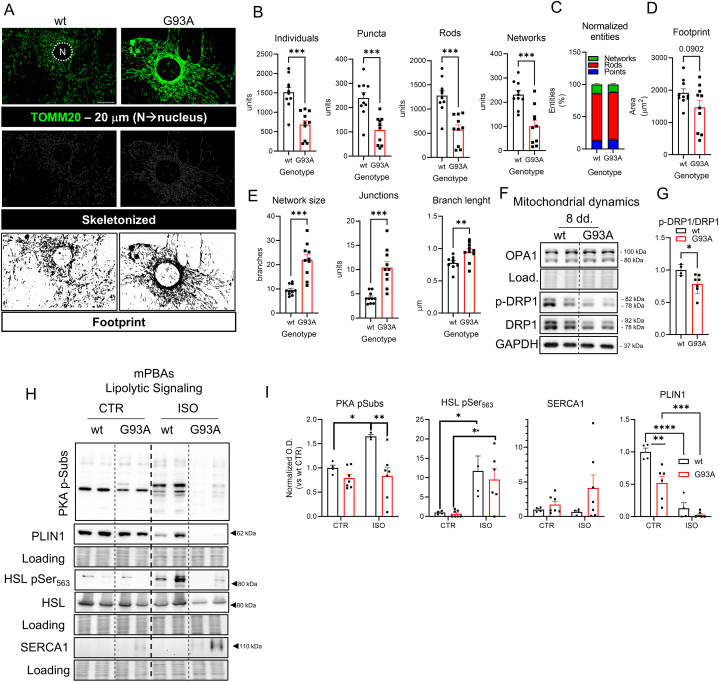
Characterization of the mitochondrial dynamics and lipolytic pathway in mPBAs from SOD1-G93A mouse model A) representative fluorescent micrographs of primary brown pre-adipocytes labelled with anti-TOMM20 and Alexafluor-488 secondary antibody. The nuclear space (N) is delimited by dotted line. Scale bar 20 μm. The skeletonized image and the footprint image were retrieved from the output of the MiNA Image J plugin. B) bar plot representing the different morphological parameters of mitochondrial individuals (individuals, rods, puncta and networks). Data are reported as mean ± SEM. Statistical significance was assessed through Student's t-test. ∗∗∗p < 0.001. C) stacked bar plot representing the percentage of mitochondrial entities (puncta, rods and networks) over total. D) bar plot representing the footprint of the total mitochondrial area expressed in μm^2^. Statistical analysis was assessed through Student's t-test. Numeric p-value was reported. E) bar plot reporting the data of the network parameters (network size, number of junctions and branch length). Data are represented as mean ± SEM. Statistical analysis was performed through Student's t-test. ∗∗p < 0.01 ∗∗∗p < 0.001. F) representative Western blot of proteins involved in the mitochondrial dynamics DRP-1 p-Ser_616_, DRP-1 and OPA-1. Loading control is shown as Ponceau Red staining and GAPDH protein levels. G) bar plot reporting the densitometric quantitation of DRP-1 pSer_616_ normalized versus total protein levels of DRP-1. Data are reported as mean ± SEM. Statistical analysis was performed through Student's t-test. ∗p < 0.05. H) representative Western blot of mPBAs from wild type and SOD1-G93A mouse models, treated or not with isoproterenol (10 μM, 16h). Lipolytic signaling proteins (PKA p-Substrates, PLIN1, HSL p-Ser_563_ and HSL) and alternative thermogenesis (SERCA1) were analyzed. Ponceau Red staining was used as loading control. I) bar plot representing the densitometric quantitation of western blots shown in panel P. Data are presented are mean ± SEM and normalized versus wild type CTR group. Statistical significance was assessed by Two-Way ANOVA with multiple comparisons correction. ∗p < 0.05; ∗∗p < 0.01; ∗∗∗p < 0.001.

The data shown in Fig. 2 highlighted the presence of mitochondrial damage, impacting on both cellular differentiation and metabolism. To get deeper information about the status of the mitochondrial network, we performed confocal microscopy and MiNA analysis on mPB pre-adipocytes (Fig. 3A). Analysis of the skeletonized network showed reduction of mitochondrial individuals, as either puncta or rods (Fig. 3B). Moreover, a reduction of the number of networks was highlighted by the analysis, although the distribution of the entity type was similar between wild type and SOD1-G93A pre-adipocytes (Fig. 3B–C). Notwithstanding, mitochondrial footprint analysis does not show a reduction in total mitochondrial area, suggesting that such alterations in network and entities abundance is not correlated to a reduction in mitochondrial mass, as also suggested by Western blot analysis of TOMM20 on differentiated mPBAs (Fig. 2G). Interestingly, mitochondrial network analysis showed an increased network size, correlating with increased number of junctions and branch length (Fig. 3E). This result prompted us to hypothesize that mitochondrial network undergoes an increased fusion process in the SOD1-G93A context. To test this hypothesis, we assessed the expression of DRP1 and OPA1 proteins in cell lysates of mPBAs at terminal differentiation (Fig. 3F–G). Results show that DRP-1 phosphorylation on Ser-616 is reduced in mPBAs from SOD1-G93A mouse model. In parallel no consistent changes in OPA1 levels was observed. We hypothesize that such changes in the mitochondrial dynamics could also impact on the cellular competence to respond to pro-lipolytic stimuli. To assess the activation of the phosphorylation cascade driven by protein kinase A (PKA), under control of the adrenergic beta-3 receptor, we used an antibody directed against the PKA target epitope (RRXS∗/T∗). In addition, we analyzed the phosphorylation of the hormone-sensitive lipase HSL, a direct target of PKA on the activating Ser_563_. We take advantage of isolated mPBAs under stimulation with isoproterenol (ISO 10 μM, 16h), a well-known agonist of beta-adrenoreceptor [44]. Western blot analysis results show that SOD1-G93A mPBAs have a defective induction of the lipolytic cascade downstream to PKA, since SOD1-G93A cells are unable to induce PKA-substrates phosphorylation under ISO treatment, in respect to wild type cell. In contrast, phosphorylation of HSL Ser_563_ appears unchanged between wild type and SOD1-G93A mPBAs under ISO stimulation (Fig. 3H–I). This discrepancy could indicate that activation of PKA and HSL phosphorylation could be regulated by different kinetics. In fact, terminal lipolysis, assessed by the levels of PLIN1, clearly show that both cell types are competent in degrading lipid droplets under stimulation, although at the basal level, SOD1-G93A cells have a reduced lipid content and lower PLIN1 levels (Fig. 3H–I), as also shown in Fig. 2A–D. Interestingly, SOD1-G93A mPBAs clearly show an increase in SERCA1 protein expression under ISO stimulation (Fig. 3H–I), still confirming that these cells are more prone to activate the alternative thermogenic program as suggested by the multi-omics analysis. Overall, the data shown above, clearly highlight defects in primary brown adipocytes from SOD1-G93A mice at different levels, suggesting that alterations observed with the multi-omics characterization are imputable to cell-autonomous impairments due to the diseased context.

### 3.4 Extracellular vesicles from SOD1-G93A BAT show alterations in size distribution and cargo

**Fig. 4:**
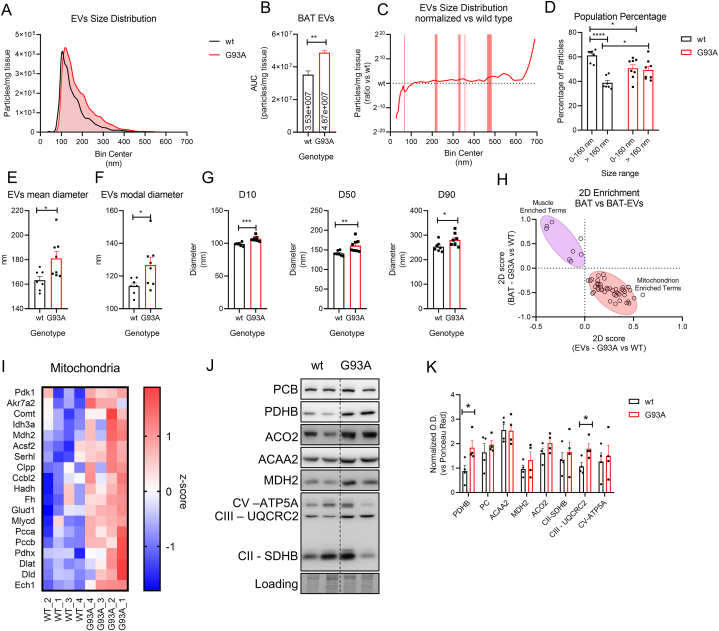
Characterization of BAT-EVs A) distribution plot of BAT-EVs size from wt and SOD1-G93A mouse models. Data are presented as particles, normalized per unit of weight of BAT tissue. B) bar plot representing the BAT-EV concentration calculated as area under curve (AUC) from panel A. Data are presented as mean ± SEM. Statistical significance was assessed by Student's t-test corrected for AUC analysis. ∗∗p < 0.01. C) distribution plot of BAT-EVs size, normalized versus the wild type group. Statistical significance between size bins was estimated by multiple *t*-test assuming gaussian distribution and homoscedasticity, without multiple comparisons correction. Statistically significant bin ranges are highlighted in red. D) bar plot representing the quantitation of population percentage of BAT-EVs from wild type and SOD1-G93A in the 0–160 nm and >160 nm ranges. Statistical significance was assessed by Two-Way ANOVA with multiple comparisons correction. ∗p < 0.05; ∗∗∗p < 0.001. E-G) bar plot representing the EVs mean and modal diameter and the D10, D50 and D90 parameters. Data are presented as mean ± SEM. Data were analyzed by Student's t-test. ∗p < 0.05; ∗∗ p > 0.01; ∗∗∗p < 0.001. H) dispersion plot representing the 2D-Enrichment analysis of BAT tissue and BAT-EVs. The two groups of muscle-related and mitochondrial-related entities are highlighted in purple and red, respectively. I) heatmap representing the z-scored values of mitochondrial proteins from the proteomics analysis of BAT-EVs from wild type and SOD1-G93A mouse models. J) representative Western blot analysis of BAT-EVs from wild type and SOD1-G93A mouse models. Mitochondrial proteins and ETC complexes were analyzed. Ponceau Red staining was used as loading control. K) bar plot representing the densitometric quantitation of Western blot in panel O. Data are reported as mean ± SEM of normalized OD value versus loading control. Data were analyzed with Student's t-test. ∗p < 0.05.

The results shown above demonstrate that BAT and mPBAs undergo molecular rearrangements leading to metabolic and phenotypic defects. The homeostasis of BAT has been shown to be strictly linked to its capacity to modulate EVs secretion to overcome physiological alterations, and this topic could be of great interest to better understand the pathological modifications occurring in ALS. We asked whether also in ALS, BAT-EVs production could be altered. To address this question, we performed EVs isolation through differential ultracentrifugation and analyzed the size distribution with Nanoparticle Tracking Analysis. Results show a positive shift in size distribution frequency and area under curve (AUC) analysis, reporting an increased EVs secretion from SOD1-G93A BAT accounting to 4.87 x 10^7^ particles/mg tissue versus 3.53 x 10^7^ particles/mg tissue of wild type BAT (Fig. 4A–B). To assess whether particular size range were differentially represented in SOD1-G93A BAT-EVs, we performed data normalization versus the wild type counterpart. Data reported in Fig. 4C show a significant decrease of vesicles with diameter between 66 and 69 nm, while increasing with diameter between 213 and 225 nm, 325–335 nm, 355–358 nm and 462–484 nm (Fig. 4C). Further data analysis confirmed that wild type EVs have lower number of particles with diameter greater that 160 nm, in respect to particles ranging 0–160 nm, while SOD1-G93A particles have lower amount of particles in the 0–160 nm range and increased abundance in the >160 nm interval (Fig. 4D). Overall, EVs mean diameter increases from 163.2 ± 3.080 nm in wild type to 181 ± 16.27 nm in SOD1-G93A, while modal diameter increases from 113.9 ± 1.857 nm in wild type to 126.7 ± 4.8 in SOD1-G93A EVs nm (Fig. 4E–F). D10, D50 and D90 parameters respectively represent the percentage of vesicles with diameter lower than the indicated. The increase in all the three parameters strengthened the information that particles from SOD1-G93A BAT mice have an increase in size (Fig. 4G). To better understand whether the pathological context could also impact on the EVs cargo, we performed proteomics analysis on total EVs lysates. Correlation plot and PCA analysis did not show a significant segregation of wild type versus SOD1-G93A samples (Supp. Figs. 3A–B). These results indicate that there are not consistent changes at the proteome level to justify a differential expression analysis. Although, to get further information about the differential regulation of the proteomics profile between the intracellular and the extracellular environment, we performed 2D enrichment on BAT proteomics versus BAT-EVs proteomics (Fig. 4H). 2D enrichment analysis showed negative correlation between BAT and BAT-EVs proteomes, with mitochondrial proteins occupying the lower-right quadrant, which represents entities underrepresented in the intracellular milieu while being overrepresented in the EVs compartment (Fig. 4H). Heatmap representation of all mitochondrial proteins identified in EVs, clearly show enrichment in SOD1-G93A EVs (Fig. 4I). To confirm the proteomics data, we performed Western blot analysis of total EVs lysates. As suggested, PDHB is one of the most upregulated proteins (Fig. 4J–K). Interestingly, also ETC complex III subunit UQCRC2 significantly increases suggesting that ejection of mitochondrial proteins through the EVs compartment could also involve proteins from the inner mitochondrial membrane (Fig. 4J–K). Overall, the reported data suggest a modulation of the vesicular secretory function of BAT in the SOD1-G93A context, with particular relevance to the selective secretion of mitochondrial particles.

### 3.5 BAT-EVs from SOD1-G93A mice modulate differentiation and homeostasis of C2C12 murine myoblasts

**Fig. 5:**
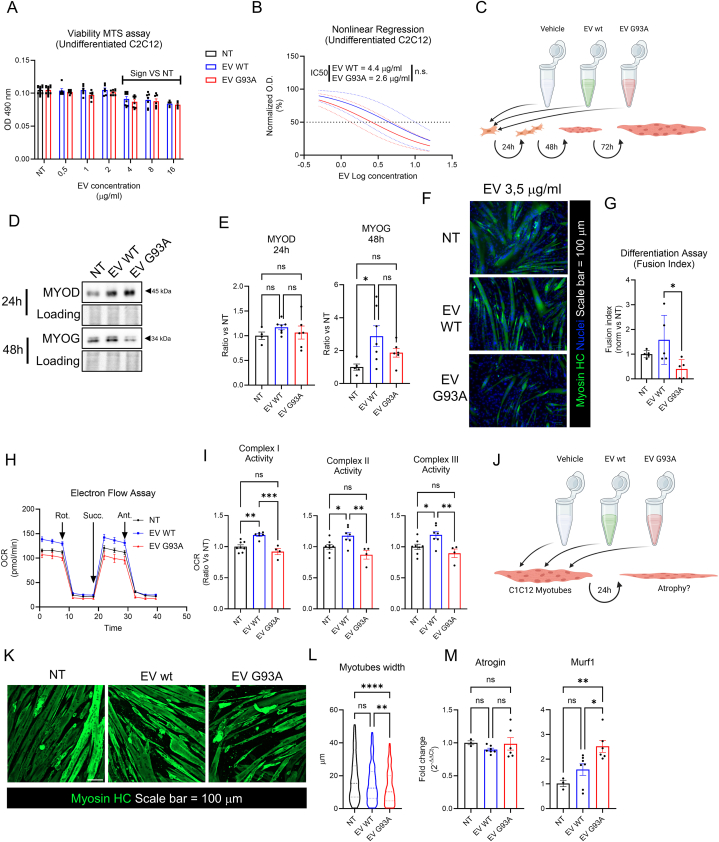
Effect of BAT-EVs on C2C12 murine myoblasts A) Bar plot representing the MTS viability assay on C2C12 myoblasts treated with BAT-EVs from wild type and SOD1-G93A mice. O.D. at 490 nm were normalized versus non-treated (NT) samples. Data are reported as mean ± SEM. Statistical significance was assessed through Two-Way ANOVA with multiple comparison correction. n = 6. B) curve fitting of data in panel A. O.D. was normalized between 0 (minimum value) and 100 % (maximum value), EV concentration was logarithmized and fitting was performed through non-linear regression analysis using a dose-response inhibitory model with variable slope. Dotted lines correspond to 95 % confidence intervals. IC50 was returned by the software and statistical significance was assessed by Student's t-test. C) cartoon representing the experimental model of C2C12 murine myoblasts treated with BAT-EVs at time zero of differentiation. The difference process was monitored at 24, 48 and 72 h after treatment. Artwork was made with Biorender https://www.biorender.com/. D) representative Western blot of C2C12 cells at 24 and 48 h after treatment with BAT-EVs. MyoD was assessed at 24 h. Myogenin was assessed at 48 h. Ponceau Red staining as loading control. E) bar plot representing the densitometric quantitation of Western blot in panel D. Data are reported as mean ± SEM and OD of bands were normalized versus the NT sample. Statistical significance was assessed by One-Way ANOVA with multiple comparisons correction. ∗p < 0.05. MyoD: n_NT_ = 4; n_WT_ = 7 n_G93A_ = 6. Myogenin: n_NT_ = 5; n_WT_ = 7; n_G93A_ = 6. F) representative fluorescent micrographs of C2C12-derived myotubes at 72 h after treatment. Sarcomeric myosin was labelled with anti-MHC (Mf-20 clone). Nuclei were counterstained with Hoechst 33342. Scale bar 100 μm. G) bar plot representing the fusion index calculated for experiment in panel F. Fusion index is expressed as percentage of myonuclei over total number of nuclei per field. Data were normalized versus NT sample. Data are reported as mean ± SEM. Statistical significance was assessed by One-Way ANOVA with multiple comparison correlations. ∗p < 0–05. n_NT_ = 5; n_WT_ = 5; n_G93A_ = 5. H) graph representing the oxygen consumption rate (OCR) of undifferentiated C2C12 cells treated with BAT-EVs, during the electron flow experiment in Seahorse XF assay. Mitochondria were stimulated with rotenone (complex I inhibitor), succinate (Complex II activator) and antimycin A (complex III inhibitor). Data are expressed in pmol O_2_/min. I) bar plots representing the electron transport chain complexed (ETC) activity assessed by the electro flow in panel H. Data are expressed as OCR and normalized versus the NT sample. Statistical significance was assessed by the One-Way ANOVA test with multiple comparisons correction. ∗p < 0.05 ∗∗p < 0.01 ∗∗∗p < 0.001. J) cartoon representing the experimental model of C2C12-derived myotubes treated with BAT-EVs. Artwork was made with Biorender https://www.biorender.com/. K) representative fluorescent micrographs of C2C12.derived myotubes treated with BAT-EVs. Sarcomeric myosin was labelled with anti-MHC (Mf-20 clone). Scale bar = 100 μm. L) violin-plot representing the myotubes width calculated from experiment in panel K. Myotubes width was calculated with an Image J dedicated plugin and expressed in μm. Data derive from single myotubes analyzed from 6 different images (one per sample in the center of the well). Statistical significance was assessed through Kruskal-Wallis test for non-normal distributions with multiple comparison correction. Dashed line represents median values. Dotted lines represent 1st and 3rd quartiles. ∗∗p < 0.01 ∗∗∗∗p < 0.0001. n_NT_ = 274; n_WT_ = 368; n_G93A_ = 318. M) bar plot representing the RT-qPCR analysis of Atrogin and Murf1 genes upon treatment with BAT-EVs for 24 h. Data were analyzed with the 2^−ΔΔCt^ method and reported as mean ± SEM versus NT sample. Statistical significance was assessed through the One-Way ANOVA test with multiple comparison correction. ∗p < 0.05 ∗∗p < 0.01. n_NT_ = 3; n_WT_ = 7; n_G93A_ = 6.

The skeletal muscle compartment is the most affected in ALS. BAT and skeletal muscle can communicate through both soluble factors and EVs and this communication can be altered in pathological conditions as metabolic syndromes and Type 2 Diabetes [25]. Moreover, it has been shown that BAT-EVs from altered environment (as cold-exposure [20]) can perturb the homeostasis of recipient cells. The prominent metabolic features of the skeletal muscle tissue in ALS [[45], [46], [47]] sustain the hypothesis that these aberrancies could also depend on factors external of the skeletal muscle tissue, that can influence its homeostasis through inter-organ communication. Given this premise, we hypothesized that an aberrant crosstalk between BAT and skeletal muscle is occurring in the SOD1-G93A mouse model. We started our investigation with a study about the capability of BAT-EVs on modulating C2C12 murine myoblasts viability. BAT-EVs from both wild type and SOD1-G93A mouse models are able to decrease C2C12 viability at a concentration over 4 μg/ml (Fig. 5A). Non-linear regression analysis allowed the calculation of the IC50 for both BAT-EVs types, accounting for 4.4 μg/ml for wild type and 2.6 μg/ml for SOD1-G93A BAT-EVs (Fig. 5B). We decided to follow up our experiments in condition of a mean dose of BAT-EVs, relatively to the IC50 calculated in Fig. 5B (3.5 μg/ml). We next tested the effect of BAT-EVs on C2C12 myoblasts differentiation, by treatment at T0 in differentiation medium and by analyzing the expression of myogenic transcription factors (MyoD and myogenin at 24 and 48 h after treatment) and end-point differentiation, at 72 h (Fig. 5C). Results shown in Fig. 5D–E show that MyoD does not change consistently between samples, while myogenin significantly increases in samples treated with BAT-EVs from wild type animals, and such increase is not recapitulated by SOD1-G93A BAT-EVs (Fig. 5 E) suggesting that wild type BAT EVs could stimulate the early phase of myogenic differentiation. BAT-EVs from SOD1-G93A mouse models were able to inhibit C2C12 terminal differentiation, as calculated by the fusion index analysis, in respect to the samples treated with BAT-EVs from wild type mice (Fig. 5F–G). Given that the myogenic differentiation correlates with an increase of the mitochondrial performance we performed the electron flow assay by using the Seahorse technology that assesses the ETC complex enzymatic activity (Fig. 5H). Complex I, II, and III-dependent oxygen consumption rate is increased in the wild type BAT-EVs-treated group both in respect to the NT and the SOD1-G93A BAT-EVs-treated group (Fig. 5 I). These data suggest that BAT-EVs can modulate the homeostasis of murine myoblasts during the differentiation process, by stimulating the early transcriptional myogenic cascade and the mitochondrial performance. Finally, we tested the effect of BAT-EVs on differentiated C2C12-derived myotubes (Fig. 5 J). To this aim we treated myotubes with 3.5 μg/ml of BAT-EVs. Results show that SOD1-G93A BAT-EVs are capable of inducing myotube atrophy (Fig. 5 K), as assessed by the calculation of myotube diameter (Fig. 5 L). Consistently, such phenotypical alteration parallels with increased expression of E3-ubiquitin ligase Murf1 but not of Atrogin (Fig. 5 M), suggesting a different modulation of the two genes by SOD1-G93A BAT-EVs.

## 4 Discussion

Among the most peculiar clinical manifestations of ALS, thermoregulation has been one of the most debated by the scientific community. Indeed, patients can differently report about feeling hot or, conversely, being unable to warm up. In animal models, it has been shown that SOD1-G93A mice have thermoregulatory defects, being unable to maintain the body temperature upon fasting, cold exposure or morphine injection [[48], [49], [50]]. Considering this evidence, mouse models suggest that thermoregulation stands for a defective physiological system in ALS pathology. Consistently, thermoregulation strictly relies on the systemic energetic balance and homeostasis, shown to be altered in ALS patients and mouse models [51]. In our study, we applied a multi-omics approach for investigating the perturbations occurring into BAT from ALS SOD1-G93A mouse model at symptomatic stage. Interestingly, alternative thermogenic mechanisms are highly upregulated and rely on the increased number of genes and proteins classically known to play an active role in the skeletal muscle homeostasis, namely, the acto-myosin complex and the sarco-endoplasmic reticulum calcium ATPases (SERCA) pumps. Interestingly, brown adipocytes have an ontogenic link with skeletal muscle cells, since multipotent Pax3^+^/Pax7^+^/Myf5^+^ stem cells provide progenitors for both the BAT and the skeletal muscle development during embryogenesis [52]. Recently, alternative thermogenic programs were described in several reports. Aquilano and colleagues [53] showed that dietary modulation consisting in low-protein/high-carb diet was able to induce thermogenic pathways relying on futile cycles as the upregulation of acto-myosin proteins and SERCA pumps in beige adipocytes. Despite, acto-myosin complexes do not provide contractile properties to adipocytes but increase heat generation through futile cycle of ATP hydrolysis [54]. SERCA pumps are essential to control calcium homeostasis and balance between cytosol and endoplasmic reticulum reservoir. SERCAs pump back calcium into the ER lumen through active transport, consuming ATP. Moreover, several mechanisms, as sarcolipin and phospholamban overexpression, uncouple ATP hydrolysis from calcium uptake, thus providing another futile cycle for heat dissipation [55]. These alternative thermogenic mechanisms could be important in physio-pathological situations where mitochondrial functionality is impaired, and BAT needs to redirect heat dissipation to non-mitochondrial systems. Generally, we refer to these mechanisms as “futile cycles”, that are defined as all those mechanisms that consume ATP without conferring a proper function to the cell. Adipose tissue, both white and brown, can trigger futile cycles in different conditions, such as: 1) the endogenous mitochondrial UCP1-independent uncoupling, mediated by the AAC protein in BAT [56,57]; 2) the creatine-dependent ADP/ATP cycling mediated by both AAC protein and creatine kinase (CK) in beige fat [58,59]; 3) the glycerolipid-free fatty acid cycle [60,61] and 4) the glyceroneogenesis-lipid cycle, one of the very first discovered in the late 70s [62]. The activation of such alternative genes could be dependent on the unaltered response by UCP1 gene/proteins, revealed in our study. The body of evidence reporting UCP1 upregulation are the ones from Ciccarone and colleagues [63] and Bayer and colleagues [64]. Ciccarone and colleagues analyzed SOD1-G93A at end-stage of the disease (150 d.p.p.), finding upregulation of UCP1 protein both in WAT and BAT. Moreover, they found upregulation of mitochondrial and lipid metabolism enzymes. In the work by Bayer and colleagues, the focused their attention of the signaling of PGC1a, a well-known inducer of oxidative metabolism and mitochondrial biogenesis [65,66]. In their analyses they found upregulation of *Ucp1 mRNA* at 130 d.p.p. in total BAT and skeletal muscle extracts. In contrast, in *ex vivo* cultured primary brown adipocytes they found increase of *Ucp1 mRNA* levels only upon treatment with 1 μM of norepinephrine, while no consistent upregulation was evidenced between wild type and SOD1-G93A cells at resting state. It has to be noted that the Authors did not report any data about UCP1 protein level and only the mRNA data can be confounding as has been finely reviewed [67]. By contrast, inconsistent modulation of *Ucp1* gene was reported in other studies. Dupuis and colleagues, in a very seminal study about defective energy metabolism in an ALS mouse model (SOD1-G86R), although finding many metabolic alterations, did not evidence upregulation of *Ucp1 mRNA* at 75- nor at 105 d.p.p., corresponding to the onset and the early symptomatic stage [48]. Consistently, the study by Steyn and colleagues, investigated other aspects regarding BAT activation [45]. In particular, they assessed BAT mass from the pre-symptomatic (50 d.p.p.) to the end-stage (150 d.p.p.) of the SOD1-G93A mouse model, recapitulating our results showing no consistent variation in BAT mass normalized per body weight. Moreover, since the variation of BAT mass can confound about the physiological status of the organ, Steyn and colleagues performed, for the first time, a positron-emission tomography (PET) coupled to injection of 18^F^-fluorodeoxyglucose (FDG) as a tracer of glucose uptake to sustain BAT metabolism upon thermogenic activation. In this experiment, there has been evidenced no increase in the FDG uptake by BAT at any stage and highlight that, at the resting level, BAT from SOD1-G93A mouse model does not increase its canonical thermogenic activity. Since, as discussed above, the body of evidence about UCP1 expression and BAT physiology was inconsistent in literature, in our study, we decided not to limit our attention to merely controlling the expression of *Ucp1* mRNA or protein. Instead, we adopted a multi-omics strategy involving both RNA-Seq and proteomics to get a deeper insight into the molecular rearrangements of this tissue in the ALS disease. Our results recapitulate the latest data discussed about the inconsistent alteration of *Ucp1* gene levels supporting the idea that canonical thermogenesis is not occurring in this context. Moreover, it has to be taken into account that ALS-related mouse models are characterized by a high heterogeneity of symptoms and pathological features that can introduce confounding factors, mostly in regard of the disease staging and animal life expectancy that varies across models, target genes and genetic mutations. Here, we propose, and show supportive data, that the mechanism could rely on alternative UCP1-independent routes. Moreover, our data show that the activation of alternative thermogenic program came with a strong downregulation of genes and proteins related to the mitochondrial compartment. We observed drastic reduction in the protein levels of pyruvate dehydrogenase isoform A and B, enzymes involved in the critical step of conversion of pyruvate to acetyl-CoA in the mitochondria, for entering the TCA cycle. PDH activity is important for maintaining energy homeostasis in ALS, as knockdown of pyruvate dehydrogenase kinase 2 (PDK2), that inhibits PDH, is able to support mitochondrial metabolism in SOD1-G93A rat model, by ameliorating the interplay between astrocytes and motor neurons [68]. In light of this, PDH expression could represent a hub for the regulation of energy metabolism in ALS, also in peripheral tissues.

In the present study, we showed that *ex vivo* cultured mPBAs have differentiation and metabolic defects. The decrease in adipocyte area is partly in accordance with Ciccarone et al. [63], that showed this reduction in both BAT and subcutaneous WAT at the end-stage (150 d.p.p.). The modulation of terminal adipogenesis correlates with the differential modulation of PPARγ isoforms. Indeed, in our study, mPBAs from SOD1-G93A mouse model have impaired expression of the PPARγ2 isoform. PPARγ2 is highly expressed in browning-resistant fat depots, strengthening the information that brown adipocytes from SOD1-G93A mice has an impairment in the induction of the canonical thermogenic program [69]. The differentiation defects came with metabolic defects in mPBAs from SOD1-G93A mouse model. Metabolic defects are a typical hallmark of ALS in different tissues and cell types. In particular, the skeletal muscle of ALS mouse model shows alteration in fiber types (glycolytic-to-oxidative switch) and in mitochondrial functionalities, that predate the onset of motor symptoms [46,[70], [71], [72]]. Notably, mitochondrial metabolism alterations have also been observed in ALS patients’ skeletal muscle samples [72,73]. Also, the neuronal compartment has metabolic defects in the ALS context. In mouse model, both spinal and cortical neurons have hampered basal respiration, maximal respiration, spare capacity and ATP production [74]. This alteration has been linked to defects in the autophagic process and in signaling pathways controlling energy homeostasis [74,75]. Adipose tissue has been poorly investigated in ALS; different reports highlight that also white adipose tissue has metabolic defects. Steyn et al., showed that epididymal AT from SOD1-G93A mice have increased lipolytic efflux and reduced fat mass, also correlated with reduced plasmatic leptin concentration [45]. According to the lipolytic process, in the present work we showed that BAT have lower activation of the lipolytic signaling under control of PKA, while mPBAs are unable to properly respond to the beta-adrenergic stimulation. Importantly, metabolic homeostasis can be rewired both *in vitro* and *in vivo*, correlating with amelioration of tissue structure and ultrastructure as well as animal motor symptoms [46,74,76]. Moreover, in our study we presented a characterization of the mitochondrial dynamics in primary pre-adipocytes extracted from BAT of wild type and SOD1-G93A mice. Our analyses highlighted that SOD1-G93A pre-adipocytes have a more fused mitochondrial network, with a decreased number of organelles and increased network size. This is in favor of the role of mitochondrial dynamics in supporting the metabolism and functionality of BAT. Indeed, human brown adipocytes typically exhibit fragmented mitochondrial morphology, which supports higher levels of catabolic processes, uncoupled respiration, and thermogenesis [77]. The interplay between mitochondrial fission and increased UCP1 expression may enhance thermogenesis in these cells. Several key proteins regulate the processes of mitochondrial fission and fusion. Dynamin-related protein-1 (DRP1), a cytoplasmic GTPase, plays a crucial role in this regulation; inhibition of DRP1 disrupts adrenaline-induced thermogenesis, underscoring its importance [77]. Exposure to cold triggers norepinephrine secretion, which leads to rapid mitochondrial fragmentation via PKA-dependent phosphorylation of DRP1, accompanied by lipolysis and UCP1 induction [78]. In beige adipocytes derived from human mesenchymal stem cells, DRP1-mediated fission enhances uncoupling activity [77]. Additionally, brown fat-specific deletion of the inner mitochondrial membrane fusion protein optic atrophy 1 (OPA1) results in mitochondrial dysfunction and impaired cold-induced thermogenesis [79]. Recent findings suggest that OPA1-dependent fumarate accumulation promotes cell-autonomous adipocyte browning [80]. Given this body of evidence, we speculate here that the mitochondrial defects highlighted by the functional analysis of extracellular flux and lipolysis competence, could be linked to the abnormal mitochondrial dynamics here observed.

Recently, our group has taken part in a study showing that physiological stimuli, as cold stress, induce mitochondrial ROS damage in BAT, which activates an alternative recycling pathway of damaged mitochondria through extracellular vesicle (EVs) release. Moreover, EVs from BAT of cold-exposed mice, had oxidized and carbonylated proteins, proxy of intracellular ROS damage. Interestingly, such EV population was capable of altering the metabolism of recipient cells, and their removal from tissue-resident macrophages was necessary for the homeostatic regulation of thermogenesis [20]. EVs have been shown to play a prominent role in ALS since they could be vehicle of misfolded proteins such as TDP-43, FUS, and SOD1 and could represent an optimal biomarker for ALS diagnosis and/or prognosis, to monitor the disease progression or to develop new therapeutical approaches [[81], [82], [83]]. The importance of studying EVs in ALS pathology is based on the fact that these vesicles are able to cross the blood brain barrier [84]. It is therefore not to be excluded that also EVs from peripheral organs pivotal involved in the ALS phenotype. In the case of EVs from peripheral organs, adipose-derived stem cells (ADSCs) and mesenchymal stem cells (MSCs) have been investigated [[85], [86], [87]]. Interestingly, AT-derived EVs exert a neuroprotective and anti-neuroinflammatory effect both in *in vitro* and *in vivo* models, not only in ALS, but also in other neurodegenerative diseases [[88], [89], [90], [91], [92]]. In particular, regarding ALS, AT-derived EVs are able to normalize the levels of aggregated SOD1 protein and reactivate CREB and PGC1alpha pathways, that ameliorate the mitochondrial phenotype [93]. Moreover, AT-derived EVs can also be repeatedly administered to the SOD1-G93A mouse model, both intravenously and intranasally, being safe for the animal and able to improve motor performance and protecting both nerves and muscles from degeneration [91]. In WAT and heart tissue mitochondrial extrusion and the interplay with immune system cells was pivotal for maintaining metabolic and inflammatory homeostasis [[94], [95], [96]]. Here we showed that BAT-EVs have different effect on the homeostasis of an *in vitro* model of myogenic cells, namely the murine C2C12 myoblasts. The interplay between BAT and skeletal muscle has been already documented, although preliminary and contradictory, in part. The evidence in support of a positive role of AT-derived EVs, show that adipocytes subjected to mitochondrial stress release EVs containing respiratory competent mitochondrial particles that can be taken-up by cardiomyocytes to support cardiac cells in the ischemic/reperfusion injury model [97]. On the contrary, AT-derived EVs could also be a source of cytokines that can regulate skeletal muscle homeostasis at different levels, perturbing satellite cell activation, myoblast proliferation/differentiation and protein catabolism as well. Such effects could be positive or detrimental on the skeletal muscle cell, depending on the type of cytokine and on the systemic status of the individual (lean/obese, sedentary/exercised, young/aged) [26]. Among these, resistin, released by AT via EVs [98], impairs myoblast fusion by activating NF-κB or reducing desmin and myogenin expression [[99], [100], [101]]. EV cargo also includes miRNAs (e.g., miR-27a, miR-130b) that induce insulin resistance and alter lipid metabolism by inhibiting PPARγ and PGC1α [102,103]. Mitochondrial Complex I (NDUFB8) loss of activity has been shown to be linked to the ALS phenotype both in animal models and patients [46,104,105]. Here we show that wild-type EVs promote Complex I protein induction during early myogenic differentiation, a process hindered by SOD1-G93A EVs, which also dampen enzymatic activity. Complex I supports differentiation via p53 acetylation and NAD+/NADH regulation, essential for mitochondrial biogenesis and myogenesis [106,107].

Consistently, we also provided data that SOD1-G93A EVs can induce atrophy on C2C12-derived myotubes. It has been shown that BAT secretes myostatin upon simvastatin treatment [108] or IRF4 loss, with IRF4 expression influenced by BAT thermogenic activation [109]. AT-derived EVs benefit skeletal muscle trophism, protecting against ischemic degeneration and enhancing regeneration in a hindlimb ischemia model via increased MyoD, Myf5, and Pax7 expression [110].

In the case of ALS, it needs to be further investigated whether also BAT-EVs could play a prominent role in regulating the disease outcome and/or development. Here, we speculate that BAT-EVs are not the main source damage for the skeletal muscle system in ALS but can contribute in part. We think that this aspect is suggested by the observation that BAT-EVs from wild type or SOD1-G93A models have different biological properties on C2C12 cells. While BAT-EVs from healthy animal could play a neuroprotective role, this could be not the case of SOD1-G93A model, as we have shown here that their proteomic profile undergoes a radical transformation.

## 5 Conclusions and limitations

Overall, our study pointed out seeding information about the alteration of the BAT in the ALS mouse model SOD1-G93A context. We showed that this tissue undergoes a drastic molecular rearrangement at the symptomatic stage of the mouse model disease, and that such alterations came with metabolic, phenotypical and secretory alterations of the brown adipocytes. Although, it remains speculative that such alterations could be imputed to BAT developmental defects. According to this point, literature data and our results are supportive of an impairment of BAT during the manifestation of the ALS phenotype, rather than to an impaired development. In fact, literature data always show alteration during the diseases state, while in the pre-onset state no changes were observed. Dupuis et al. [48] do not see changes at 75 or 105 d.p.p., Steyn et al. [45] analyzed from 50 to 150 d.p.p., Bayer et al. [64] analyzed BAT from 60 to 130 d.p.p. and Ciccarone et al. [63] at 150 d.p.p. In all these works, when evidenced, alterations were observed only at symptomatic stage. The bioactive role of BAT-derived EVs on the modulation of the homeostasis of C2C12 myoblasts and myotubes are suggestive that BAT could also play a role in skeletal muscle degeneration observed in ALS. Here, we are not speculating that BAT-EVs are prominent in determining the pathological features of skeletal muscle, but they could be an exacerbating factor. Of course, the comparison of SOD1-G93A model with other mouse models (FUS, TDP-43) is needed, since it should be cleared whether our findings could be general of the ALS disease or are specifical for the SOD1-mutation. Further investigation could be made to understand whether BAT could present pathological feature also earlier on the symptom onset. This particular body of research would clarify whether many alterations are manifested also earlier and, as other peripheral organ show, could predate the induction of motoneuron degeneration, and if it could represent a putative target organ or to have a prognostic/diagnostic significance.

## CRediT authorship contribution statement

**Marco Rosina:** Writing – review & editing, Writing – original draft, Visualization, Validation, Resources, Project administration, Methodology, Investigation, Funding acquisition, Formal analysis, Data curation, Conceptualization. **Silvia Scaricamazza:** Methodology, Investigation, Data curation. **Flaminia Riggio:** Methodology, Investigation, Data curation. **Gianmarco Fenili:** Methodology, Investigation, Data curation. **Flavia Giannessi:** Methodology, Investigation, Data curation. **Alessandro Matteocci:** Methodology, Investigation, Data curation. **Valentina Nesci:** Methodology, Investigation, Data curation. **Illari Salvatori:** Methodology, Investigation, Data curation. **Daniela F. Angelini:** Writing – review & editing, Supervision. **Katia Aquilano:** Writing – review & editing, Supervision, Conceptualization. **Valerio Chiurchiù:** Writing – review & editing, Supervision, Data curation. **Daniele Lettieri Barbato:** Writing – review & editing, Supervision, Conceptualization. **Nicola Biagio Mercuri:** Supervision, Project administration. **Cristiana Valle:** Writing – review & editing, Supervision, Funding acquisition, Conceptualization. **Alberto Ferri:** Writing – review & editing, Supervision, Funding acquisition, Conceptualization.

## Data availability

All data and reagents will be made available upon reasonable request to the Lead Contact/Corresponding Author Dr. Marco Rosina (marco.rosina@iss.it). The mass spectrometry proteomics data have been deposited to the ProteomeXchange Consortium via the PRIDE [111] partner repository with the dataset identifier PXD054147. The RNA-Seq data are available at GEO identifier GSE273052.

## Ethic declaration

All animal procedures were performed following the European guidelines for the use of animals in research (2010/63/EU) and the requirements of Italian laws (D.L. 26/2014); they were approved by the Italian Ministry of Health (protocol number 293/2021-PR).

## Declaration of competing interest

The authors declare that they have no known competing financial interests or personal relationships that could have appeared to influence the work reported in this paper.

## Appendix A Supplementary data

Multimedia component 1 Multimedia component
1

Multimedia component 2 Multimedia component
2

Multimedia component 3 Multimedia component
3

Multimedia component 4 Multimedia component
4
