# Supplementary material for: Brown Adipose Tissue undergoes pathological perturbations and shapes C2C12 myoblast homeostasis in the SOD1-G93A mouse model of Amyotrophic Lateral Sclerosis
Source: Heliyon. 2025 Jan 23;11(3):e41801. doi: 10.1016/j.heliyon.2025.e41801 (PMC11800085; doi:10.1016/j.heliyon.2025.e41801)
Supplement: Multimedia component 4 [file mmc4.docx]

Brown Adipose Tissue undergoes pathological perturbations and shapes C2C12 myoblast homeostasis in the SOD1-G93A mouse model of Amyotrophic Lateral Sclerosis.

Marco Rosina ^1,2,^*, Silvia Scaricamazza ^2,3^, Flaminia Riggio ^4^, Gianmarco Fenili ^2,5^, Flavia Giannessi ^6,7^, Alessandro Matteocci ^8,9^, Valentina Nesci ^2,10^, Illari Salvatori ^2,11^, Daniela F. Angelini ^7^, Katia Aquilano ^12^, Valerio Chiurchiù ^3,8^, Daniele Lettieri Barbato ^12^, Nicola Biagio Mercuri ^1,10,13^, Cristiana Valle ^2,3,#^, Alberto Ferri ^2,3,#^

1. Unit of Neurology, Fondazione PTV Policlinico Tor Vergata, Viale Oxford 81, 00133, Rome, Italy
2. Laboratory of Neurochemistry, IRCCS Fondazione Santa Lucia, Via del fosso di fiorano 64, 00143, Rome, Italy
3. Institute of Translational Pharmacology, National Research Council, Via del fosso del cavaliere 100, 00133, Rome Italy
4. Department of Biology and Biotechnology “Charles Darwin”, University of Roma “La Sapienza”, 00161, Rome, Italy
5. Department of Movement, Human and Health Sciences University of Rome "Foro Italico”, Piazza Lauro de Bosis 6, 00135 Rome, Italy
6. Laboratory of Molecular Virology and Antimicrobial Immunity, Department of Science, Roma Tre University, 00146 Rome, Italy
7. Neuroimmunology Unit, IRCCS Fondazione Santa Lucia, Via del fosso di fiorano 64, 00143, Rome, Italy
8. Laboratory of Resolution of Neuroinflammation, IRCCS Fondazione Santa Lucia, Via del fosso di fiorano 64, 00143, Rome, Italy
9. PhD program in Immunology, Molecular Medicine and Applied biotechnologies, University of Rome “Tor Vergata”, 00133 Rome, Italy
10. Department of Systems Medicine, University of Roma "Tor Vergata", 00133 Rome, Italy.
11. Department of Experimental Medicine, University of Roma "La Sapienza", 00161, Rome, Italy.
12. Department of Biology, University of Rome “Tor Vergata”, via della ricerca scientifica, 00133, Rome, Italy
13. Laboratory of Experimental Neurology, IRCCS Fondazione Santa Lucia, Via del fosso di fiorano 64, 00143, Rome, Italy

* Lead Contact - Correspondence to Marco Rosina (MR), [marco.rosina90@gmail.com](mailto:marco.rosina90@gmail.com)

# These authors contributed equally to this work


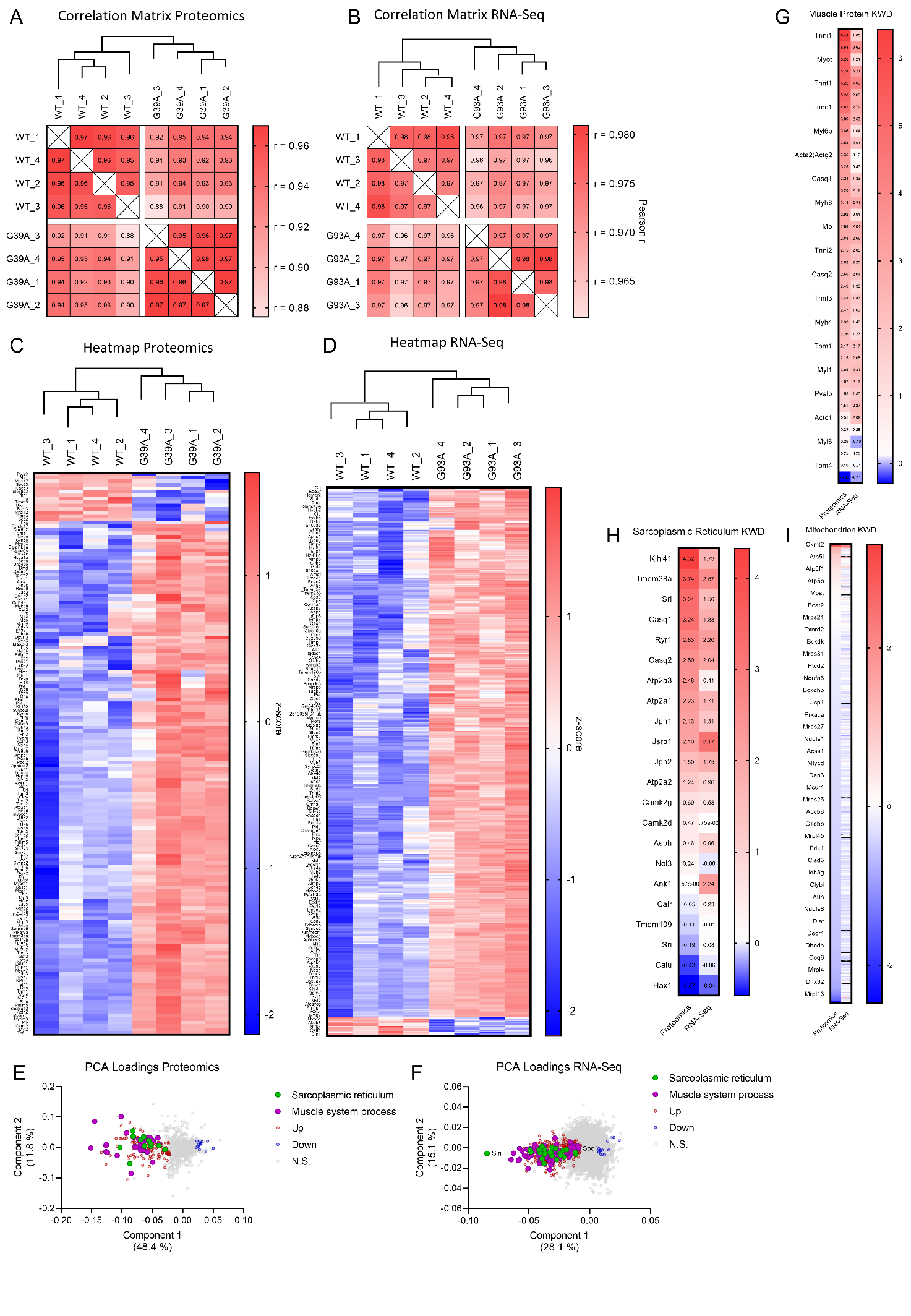


### Supplementary Figure 1: Proteomics and RNA-Seq supplementary information

A-B) heatmaps representing the Pearson correlation r values for dataset in proteomics and RNA-Seq analyses. C-D) heatmap representing the significantly regulated entities (proteins and genes, respectively) from proteomics and RNA-Seq analyses. Values are normalized in z-scoring and plotted as red (for up-regulated) and blue (for down-regulated).E-F) dispersion plots representing the distribution of entities (proteins and genes, respectively) according to Components 1 and Component 2 in the PCA analysis of Figure 1 D-E. G-H-I) heatmap representing the z-scored expression values of genes belonging to the relative Gene Ontology category. Values are plotted as red (for up-regulated) and blue (for down-regulated).


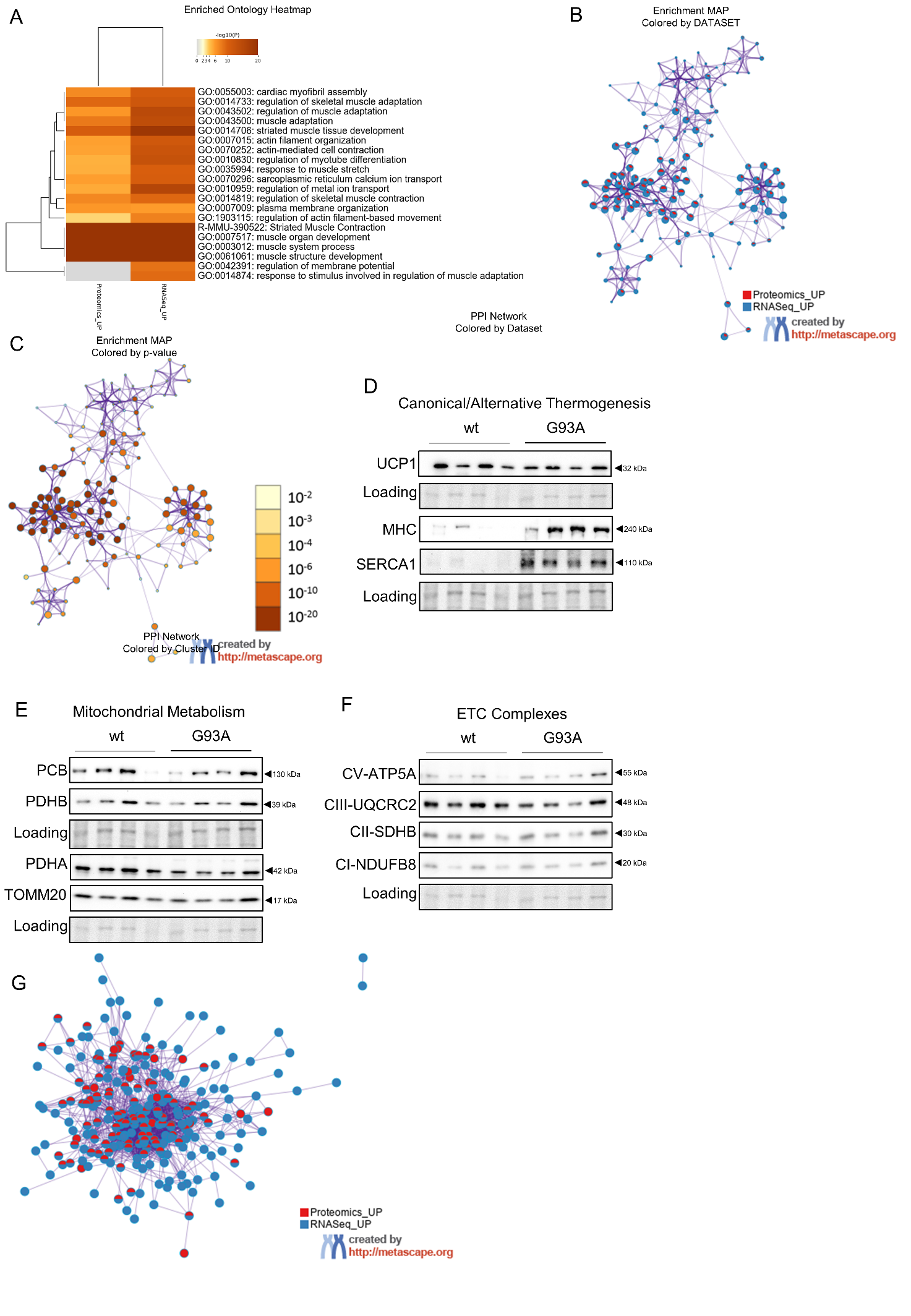


### Supplementary Figure 2: Gene Ontology supplementary information and Clustering

A) heatmap representing the enriched gene ontology -Log10 p-values in the Metascape analysis of integrated proteomics and RNA-Seq analyses upregulated entities. B) enrichment map colored by dataset (proteomics upregulated in red and RNA-Seq upregulated in blue), relative to **Fig.**  1J. C) enrichment map colored by p-value relative to **Fig.**  1J. D) representative western blot analysis of total BAT extracts for canonical (UCP1) and alternative (MHC, SERCA1) thermogenic markers. Ponceau S staining was taken as loading control. E) representative western blot analysis of total BAT extracts for mitochondrial proteins (PCB, PDHA, PDHB and TOMM20). Ponceau S staining was taken as loading control. F) representative western blot analysis of total BAT extracts for mitochondrial ETC complexes (NDUFB8, SDHB, UQCRC2 and ATP5A). Ponceau S staining was taken as loading control. G) protein-protein interaction (PPI) network, colored by dataset (proteomics upregulated in red and RNA-Seq upregulated in blue), relative to the Metascape integrated analysis.


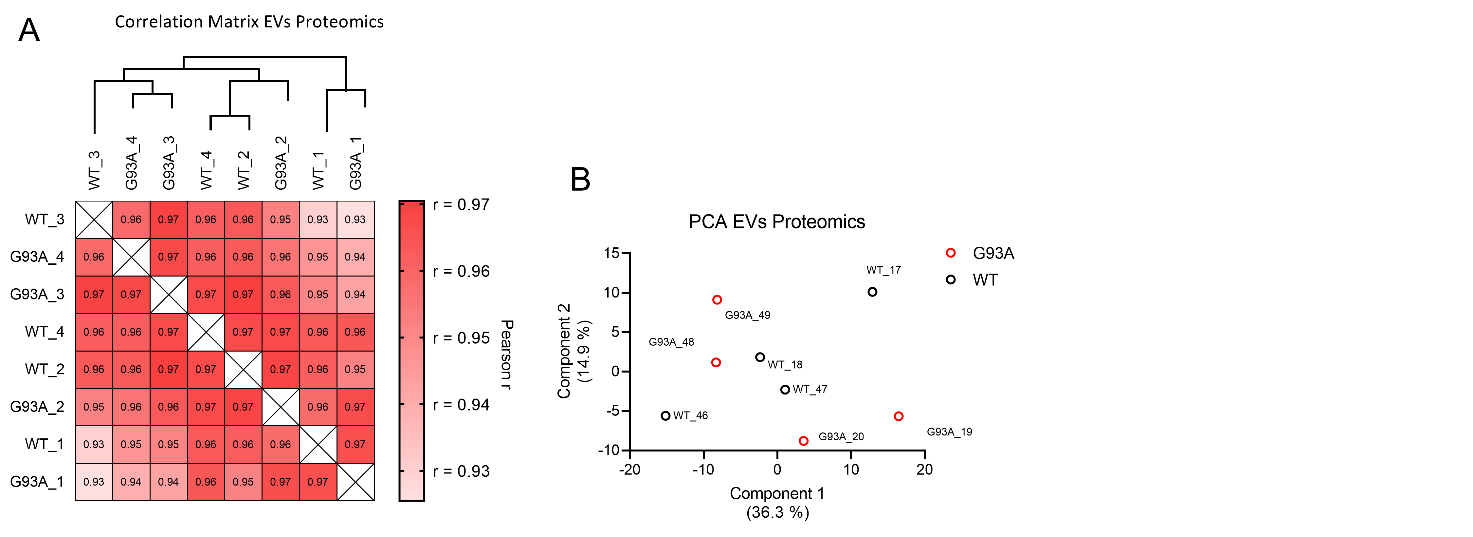


### Supplementary Figure 3: proteomics and enrichment analysis of BAT EVs supplementary information

A) heatmap representing the Pearson correlation r values for dataset in proteomics data of BAT-EVs. B) dispersion plot representing the principal component analysis of BAT EVs from wild type and G93A mouse models.
